# Supplementary material for: The effects of pre-intervention mindset induction on a brief intervention to increase risk perception and reduce alcohol use among university students: A pilot randomized controlled trial
Source: PLoS One. 2020 Sep 17;15(9):e0238833. doi: 10.1371/journal.pone.0238833 (PMC7498304; doi:10.1371/journal.pone.0238833)
Supplement: S1 Table — (DOCX) [file pone.0238833.s001.docx]

**S1 Table. Inter-correlation of variables at baseline.**

|  | (2) | (3) | (4) | (5) | (6) | (7) | (8) | (9) | (10) | (11) |
| --- | --- | --- | --- | --- | --- | --- | --- | --- | --- | --- |
| (1) AUDIT | .32** | .19* | .59** | .35** | .06 | .40** | .62** | .59** | .36** | -.25** |
| (2) Alcohol Standard Units^1^ | - | .35** | .10 | -.08 | -.17 | -.17 | .06 | .24 | -.07 | .01 |
| (3) DOSPERT |  | - | .19* | .17 | -.03 | .20* | .05 | .29** | -.04 | -.03 |
| (4) FAR-PPV^1^ |  |  | - | .62** | .29** | .54** | .65** | .69** | .44** | -.29** |
| (5) FAR-PV^2^ |  |  |  | - | .31** | .38** | .34** | .46** | .26** | -.20* |
| (6) FAR-ARP^3^ |  |  |  |  | - | .28** | .22* | .23* | .25** | -.03 |
| (7) FAR-PE^4^ |  |  |  |  |  | - | .48** | .47** | .27** | -.18 |
| (8) SOC-Recognition |  |  |  |  |  |  | - | .81** | .70** | -.31** |
| (9) SOC-Ambivalence |  |  |  |  |  |  |  | - | .59** | -.31** |
| (10) SOC-Taking Steps |  |  |  |  |  |  |  |  | - | -.38** |
| (11) URICA Precontemplation |  |  |  |  |  |  |  |  |  | - |

* p ≤ .05. ** p ≤ .01.

^1^ Alcohol Standard Units consumed in the 28 days before intervention

^2^ FAR subscale perceived personal vulnerability

^3^ FAR subscale peer vulnerability

^4^ FAR subscale affective risk perception

^5^ FAR subscale precaution effectiveness
