# Supplementary material for: The effects of pre-intervention mindset induction on a brief intervention to increase risk perception and reduce alcohol use among university students: A pilot randomized controlled trial
Source: PLoS One. 2020 Sep 17;15(9):e0238833. doi: 10.1371/journal.pone.0238833 (PMC7498304; doi:10.1371/journal.pone.0238833)
Supplement: S1 File — (PDF) [file pone.0238833.s003.pdf]

Preliminary explanatory note

The study reported here is part of a “Research Unit” (“Forschergruppe”) which is a funding instrument of the German Research Foundation for medium-term cooperative projects of six to ten independent projects with joint thematic focus.

Overview of the process of ethics approval:

1. In a first step, the Research Unit “The Dynamics of Risk Perception and Behavior in the Context of Mental and Physical Health” (RISKDYNAMICS; FOR2374) submitted a joint proposal with descriptions of the ten related subprojects to the Institutional Review Board of the University of Konstanz, which was evaluated positively on January 26, 2016 (see attached statement). This very lengthy proposal will not be reported here.

2. Subsequently, the single subprojects submitted individual proposals to the IRB as *addenda* to original proposal of the Research Unit specifying the information relevant for the evaluation of human subject studies. The subproject P8 “Addiction Risk”, submitted separate proposals for each of the planned studies; this manuscript is related to P8 Study 2 of Work Package 1. The positive statement of the IRB is dated July 19, 2016.

3. This study had to be amended in the following year: Because of different reasons the study design had to be adapted to the problem of recruitment; the study originally planned to include psychotherapy patients with risky alcohol use. The amendment was related to the inclusion of a student sample with risky alcohol use into the study.

In the following document, the proposals and statements will be shown in chronological order. A German original will be followed by an English translation. The original documents in German have a white background color. The English translations are displayed with blue background.

Universität Konstanz

Universität Konstanz Department of Biology ·Fach 657 D-78457 Konstanz

Prof. Dr. Marcel Leist

Doerenkamp-Zbinden-Chair -
in vitro toxicology and biomedicine

+49 7531 88-5037
Office +49 7531 88-5038

Marcel.Leist@uni-konstanz.de
www.cms.uni-konstanz.de

Specific project title. " *The Dynamics of Risk - Perception and Behavior in the Context of Mental and Physical Health (RISKDYNAMICS)* (FOR 2374)" to be evaluated at the DFG

RefNo: IRB16KN007

26.01.2016

To whom it may concern,

in my function as the Head of the Ethics Committee (institutional review board, IRB) of the University of Konstanz, I would like to deliver the following statement regarding the research proposal (Forschergruppe) "*RISKDYNAMICS*: *The Dynamics of Risk - Perception and Behavior in the Context of Mental and Physical Health*" submitted by Prof. Dr. Britta Renner and Prof. Dr. Harald Schupp (Speakers), and to be performed in the context of the employment of the applicant at the University of Konstanz:

"**After vetting the Description of Work (DoW), the project, with all its sub-projects has been found to comply with the Ethics Guidelines of the University of Konstanz**. Notably, the evaluation of the sub-projects (Teilprojekte, see attachment) of the Forschergruppe proposal has been limited to the identification and judgement of ethical issues. A standard evaluation considers the design of the studies, and an evaluation of specific information material prepared for the individual study (informed consent form, participant information sheet, etc.). The latter have not been evaluated. Such material will be reviewed by the IRB on an individual study basis prior to the onset of the enrollment procedure".

This judgment was made on the basis of following information and guidelines: (1) the text of the Forschergruppe proposal, submitted to the DFG on 24rd November 2015; (2) the IRB application from 09.12.2015, including an outline of the project parts and the CVs of the applicants; (3) an overview of all applied sub-projects (Annex).
National and international guidelines (in particular the WMA Declaration of Helsinki - Ethical Principles for Medical Research Involving Human Subjects) have been taken into account. The specific regulations of the University for ethical experimentation and data storage and recommendations by German and Swiss medical associations as well as societies of psychology have also been taken into account.
The University has an administrative procedure in place that will ensure that ethics guidelines as laid down in German national law and international conventions are fully respected. The Ethics Committee will ensure and, if necessary, enforce the proper adherence to ethics guidelines during and after the project.

Sincerely,

M. Leist

Prof. Dr. M. Leist

Annex: List of projects considered for IRB evaluation

P1-P8 research projects, A1 adjunct research project, Z coordination

P1	**Contagious Risk Perception**<br>The Social Dynamics of Risk Perception and Attitudes towards Preventive Behaviors<br>PI: Prof. Dr. Wolfgang **Gaissmaier**, Social Psychology & Decision Sciences<br>Aligned Projects: P2, P3, P4, P5
P2	**Media Scare**<br>Tracking Neural Dynamics of Risk Perception and Predicting Precautionary Behaviors<br>PI: Dr. Ralf **Schmälzle** & Prof. Dr. Harald **Schupp,** Biological & General Psychology<br>Aligned Projects: P1, P3, P4, P5
P3	**Risk Perception 3.0**<br>Studying Risk Perceptions and Responses to Critical Events using Social Media<br>PI: Prof. Dr. Ulf-Dietrich **Reips**, Psychological Methods & Assessment<br>Aligned Projects: P1, P2
P4	**Social Contact Risk**<br>Dynamic Changes in Social Risk Perception and Interpersonal Behavior<br>PI: Prof. Dr. Harald **Schupp**, Biological & General Psychology & Prof. Dr. Britta **Renner,** Health Psychology & Psychological Assessment<br>Aligned Projects: P1, P2, P5
P5	**Personalized Risk**<br>Changes in Risk Perception and Health Behavior after Multiple Personalized Risk Feedback<br>PI: Prof. Dr. Britta **Renner** & Dr. Martina **Gamp**, Health Psychology & Psychological Assessment<br>Aligned Projects: P1, P2, P4
P6	**Updating Risk**<br>Mindsets, Post-decisional Defensiveness, and Dynamic Updating Processes in Risk Perceptions and Risk-Taking<br>PI: Prof. Dr. Peter **Gollwitzer**, Social Psychology & Motivation<br>Aligned Projects: P5, P7, P8
P7	**Cumulative Risk**<br>Modeling Risk Perceptions and Risk Taking in Dynamic Situations<br>PI: Prof. Dr. Ronald **Hübner**, General & Cognitive Psychology<br>Aligned Projects: P6, P8

P8	**Addiction Risk**<br>Dynamics of Risk Perception and Risk Behavior in Alcohol Addiction<br>PI: Prof. Dr. Brigitte **Rockstroh** & Dr. Michael **Odenwald**, Clinical Psychology<br>Aligned Projects: P6, P7, A1
A1	**Trauma Risk (Adjunct Project, funding provided by the EU)**<br>Modeling Risk Perceptions and Risk-Taking in PTSD Patients<br>PI: Prof. Dr. Thomas **Elbert** & Dr. Maggie **Schauer**, Clinical Psychology & Neuropsychology<br>Aligned Projects: P5, P8
Z	**Coordination, Training, & Management**<br>Prof. Dr. Britta **Renner** & Prof. Dr. Harald **Schupp**

Universität Konstanz

Universität Konstanz · Postfach 27 · 78457 Konstanz

Herrn Prof. Marcel Leist
Ethikkommission der Universität Konstanz

Dr. Michael Odenwald

Leitender Psychologe, Forschungsstation für Schizophrenie

Leiter, Psychotherapeutische Hochschulambulanz für Forschung & Lehre

Universitätsstraße 10
D-78464 Konstanz
+49 (0) 75 31 / 88 - 4621
Fax +49 (0) 75 31 / 88 - 4838

michael.odenwald@uni-konstanz.de
www.uni-konstanz.de

01.06.2016

Betr.: Nachtrag Ethikantrag Teilprojekt der Forschergruppe „The Dynamics of Risk – Perception and Behavior in the Context of Mental and Physical health (RISKDYNAMICS)"

Sehr geehrter Herr Prof. Leist,
sehr geehrte Damen und Herren,

im Rahmen der o.g. Forschergruppe bin ich zusammen mit Prof. Brigitte Rockstroh für das Teilprojekt P8 „Addiction Risk: Die Dynamik von Risikowahrnehmung und Risikoverhalten bei Alkoholabhängigkeit" verantwortlich. Das Teilprojekt besteht aus zwei unabhängigen Work Packages (WP), jedes WP beinhaltet mehrere Studien bzw. Experimente.

Für WP1 bin ich hauptverantwortlich.
Anbei sende ich Ablauf, Instruktionen und Instrumente, die in P8 WP1 Studie 2 eingesetzt werden.
In dieser Studie werden keine Medikamente verabreicht oder biologische Interventionen durchgeführt. Es geht hier um gesprächsbasierte Interventionen und Fragebögen.

Ich bitte um die Bescheinigung der ethischen Unbedenklichkeit der Studie.

Mit freundlichen Grüßen

Michael Odenwald

Dr. Michael Odenwald

Titel der Studie: Preparedness Effects in the Context of Feedback: Variation of Treatment Effects after Mindset Induction

Zum Hintergrund:
Riskanter Alkoholkonsum ist häufig und schädlich. Ärztliche Ratschläge und Kurzinterventionen sind effiziente Methoden, riskanten Alkoholkonsum zu reduzieren, jedoch ist unklar, was die Effektivität dieser Methoden beeinflusst. In dieser Studie testen wir ein experimentelles Paradigma, das Zustandsvariablen untersuchbar machen soll, die auf den Erfolg von solchen Kurzinterventionen wirken.

Zum Ablauf:
PsychotherapiepatientInnen mit riskantem Alkoholkonsum werden gebeten, an der Studie teilzunehmen (Einverständniserklärungen siehe Anhang). Sie werden in drei Psychotherapie-Ambulanzen (alle in KN) rekrutiert: an der Hochschulambulanz für Forschung und Lehre der Universität Konstanz sowie an den kooperierenden Ambulanzen der Tübinger Akademie für Verhaltenstherapie und des dgvt Ausbildungszentrums für Psychotherapie Bodensee (apb). Riskanter Alkoholkonsum wird nach Empfehlungen der WHO mittels eines validierten Kurzfragens erfasst (Barbor et al., 2001). Alle PatientInnen mit riskantem Alkoholkonsum erhalten die von der Weltgesundheitsorganisation empfohlene psychologische Kurzintervention (ASSIST-linked Brief Intervention, Humeniuk et al., 2010a; siehe Anhang). Die Studie untersucht, ob diese Intervention je nach Zustand der PatientInnen unterschiedlich wirksam ist. Der Zustand wird experimentell induziert durch eine kurze, schriftlich zu bearbeitende Aufgabe, wobei die Teilnehmer über ein zwischenmenschliches Problem und dessen Lösung nachdenken sollen (Gollwitzer, 2012; siehe Anhang). Randomisiert wird dadurch entweder der implemental oder deliberative Mindset induziert. Der Untersuchungsleiter hat dabei keine Kenntnis über die Art des induzierten Mindsets, das der Versuchsperson ein verschlossener Umschlag mit einer der beiden Induktionsaufgaben übergeben wird, mit der Bitte dies alleine zu bearbeiten. Implemental Mindset steht damit im Zusammenhang, dass jemand entschlossen ist, eine Entscheidung in die Tat umzusetzen. Deliberative Mindset bedeutet, jemand sammelt und wägt Informationen ab, bevor er/sie eine Entscheidung trifft.
Unsere Hypothese ist, dass je nach Mindset, die Bereitschaft variiert, den Ratschlag bzgl. des Alkoholtrinkens zu akzeptieren.
Die psychologische Kurzintervention wird durch trainierte und unter psychotherapeutischer Supervision stehende PsychologInnen oder MasterstudentInnen vorgenommen.

Zur Aufklärung der TeilnehmerInnen:
Die teilnehmenden Patienten wissen zunächst nicht, dass es sich um eine einzige Studie handelt; sie denken zunächst, sie nehmen an zwei verschiedenen Befragungen teil, die nichts miteinander zu tun haben. Am Ende des Studienzeitraums, nach drei Monaten, werden die Patienten über die Studie voll aufgeklärt.

Instrumente:
Im Anhang sind der Ablaufplan der Studie, die Einverständniserklärungen sowie die verwendeten Instrumente, die im folgenden kurz beschrieben werden.
AUDIT:
Der Alcohol Use Disorder Identification Test (AUDIT; Barbor et al., 2001) ist ein von der WHO entwickelter Screening-Fragebogen mit 10 Items, die mit vorgegebenen Antwortmöglichkeiten beantwortet werden. Er hat eine hohe Validität bei der Identifizierung von riskantem Alkoholkonsum.
SOKRATES:
Die Stages of Change Readiness and Treatment Eagerness Scale (SOCRATES; Miller & Tonigan, 1996) misst die Therapiemotivation bei Alkoholpatienten. Die deutsche Version hat insgesamt 19 einfache Items, die auf einer 5-stufigen Likert-Skala beantwortet werden. Dieses Instrument wird häufig in der klinischen Praxis eingesetzt. Die deutsche Version ist erprobt und gut validiert.
VSS-k:
Die Veränderungsstadien-Skala (VSS; Fecht et al., 1998) ist die deutsche Version des University of Rhode Island Change Assessments (URICA), das die Veränderungsmotivation misst. Die deutsche Kurzvesion VSS-k hat 16 Items, die auf einer 5-stufien Likert-Skala beantwortet werden. Die Subskala „Precontemplation“ misst den Zustand, in dem keine Einsicht in die Problematik gegeben ist, und hat 4 Items. Das Instrument ist gut validiert und wird in sehr vielen Sprachen eingesetzt. (Diese 4 Items werden im Anhang als letzte 4 Items des SKRATES-N aufgeführt).
FAR:
Der Fragebogen zur Alkoholbezogenen Risikoeinschätzung (FAR; Klepper et al., under revision) misst verschiedene Dimensionen der subjektiven Risikoeinschätzung von typischen Risiken, die durch den chronisch riskanten Konsum von Alkohol entstehen. Er wird hier in seiner Kurzform mit 20 Items eingesetzt, die jeweils auf einer 5-stufigen Likert-Skala beantwortet werden.
DOSPERT
Die Domain-Specific Risk-Taking Scale (DOSPERT; Blais & Weber, 2006) ist ein Fragebogen mit insgesamt 60 Items, der die generelle Bereitschaft eines Menschen misst, Risikoverhalten zu zeigen, die subjektive Einschätzung von Risiken und der erwartete Nutzen von typischen Risikosituationen. Der DOSPERT wird international häufig in der Forschung eingesetzt und gilt als das Standardinstrument.
TLFB:
Der Timeline Follow Back (TLFB; Sobell & Sobell, 1992) ist eine Kalendermethode, mit der rückblickend der Alkoholkonsum erfasst wird, indem die Standarddrinks pro Kalendertag im untersuchten Zeitraum erfasst werden. Er gilt heute als das Standardverfahren zur retrospektiven Erfassung von Alkoholkonsum und ist vielfach validiert.
ASSIST:

Der Alcohol, Smoking and Substance Involvement Screening Test (ASSIST; Humeniuk et al., 2010b) ist ein kurzes von der WHO entwickeltes Interview-Verfahren mit 8 Fragen, das von trainierten Personen in der Primary Health Care (in Deutschland Hausarztversorgung) durchgeführt werden kann. Der Summenscore gibt an, wie risikoreich der berichtete Konsum verschiedener Substanzen ist. Hier verwenden wir lediglich das Instrument im Zusammenhang mit Alkoholkonsum. Der Fragebogen ist die Grundlage der ASSIST-linked Brief Intervention (Humeniuk et al., 2010a), in der eine standardisierte therapeutische Rückmeldung des Interviewergebnisses gegeben wird.

Literatur:

Barbor, T.F., Higgings-Biddle, J.C., Saunders, J.B. & Monteiro, M.G. (2001). AUDIT – The alcohol use disorder identification test. guidelines for use in primary Care. 2nd edition. Geneva: World Health Organization.

Blais, A-R. and E.U. Weber (2006). A domain-specific risk-taking (DOSPERT) scale for sdult populations. *Judgment and Decision Making,* 1, 33-47.

Fecht, J., Heidenreich, T., Hoyer, J., Lauterbach, W., & Schneider, R. (1998). Veränderungsstadien bei stationärer Alkoholentwöhnungsbehandlung: Probleme der Diagnostik. *Verhaltenstherapie und Psychosoziale Praxis, 30*, 403-419.

Gollwitzer, P.M. (2012). Mindset theory of action phases. In P. Van Lange, A.W. Kruglanski & E.T. Higgins (Eds.), *Handbook of Theories of Social Psychology* (pp. 526-545). London: Sage Publications.

Humeniuk, R., Henry-Edwards, S., Ali, R., Poznyak, V., & Monteiro, M. G. (2010a). The ASSIST-linked brief intervention for hazardous and harmful substance use: a manual for use in primary care. Geneva: World Health Organization.

Humeniuk, R., Henry-Edwards, S., Ali, R., Poznyak, V., & Monteiro, M. G. (2010b). The Alcohol, Smoking and Substance Involvement Screening Test (ASSIST): manual for use in primary care. Geneva: World Health Organization.

Klepper, S.I., Odenwald, M., Rockstroh, B. (in revision). Risikowahrnehmung und Alkoholabhängigkeit. *Sucht*.

Miller, W. R., & Tonigan, J. S. (1996). Assessing drinkers' motivation for change: The Stages of Change Readiness and Treatment Eagerness Scale (SOCRATES). *Psychology of Addictive Behaviors* 10, 81-89.

Sobell, L. C., & Sobell, M. B. (1992). Timeline follow-back: A technique for assessing selfreported alcohol consumption. In R.Z. Litten & J. Allen (Eds.). *Measuring alcohol consumption: Psychosocial and Biological Methods* (pp. 41-72). New Jersey: Humana Press..

Anhang

1. Ablaufplan der Studie

2. Einverständniserklärung

3. Instruktionen für die Teilnehmer

4. Fragebogen-Instrumente

5. Mindset-Induktion
5.1 Deliberative Mindset
5.2 Implemental Mindset

6. ASSIST und ASSIST-linked Brief Intervention

1. Ablaufplan der Studie

Ablaufplan

1. Termin (Abklärungsgespräch):

Vor Beginn einer Psychotherapie in den beteiligten Ambulanzen findet ein 30-minütiges Abklärungsgespräch mit einer/einem erfahrenen Therapeuten/in statt.

Am Ende des Abklärungsgesprächs, wenn die Eignung für Psychotherapie festgestellt wurden und der Pat./die Pat. sich entschlossen hat, eine Psychotherapie an der Ambulanz zu beginnen, wird er/sie gefragt, ob er/sie bei der Studie mitmachen will.

Daraufhin: Ausfüllen der Einverständniserklärung, des AUDIT und des SOKRATES (Version 1), der Subskala „Precontemplation" des VSS-k, des Fragebogens zur Alkoholspezifischen Risikowahrnehmung und der deutschen Version der Domain-Specific Risk-Taking Scale.

Bei Erreichen eines AUDIT Cut-off- Wertes von 5 werden Patienten in den Interventionsteil der Studie aufgenommen und weitere Studien-Termine vereinbart neben den Terminen im Rahmen der laufenden Psychotherapie.

2. Termin:

(1) Mindset- Induktion (50% der Teilnehmer erhält den „Abwägen" Fragebogen, die anderen 50% die Instruktion „Pläne machen")

(2) Ausfüllen des Timeline Follow Back (über die letzten vier Wochen)

(3) Ausfüllen des SOKRATES (Version 2) und der Subskala „Precontemplation" des VSS-k

(4) Durchführen des ASSIST- Interviews

(5) Durchführung der ASSDIST-linked Brief Intervention

3. Termin (Standard-Diagnostik der Ambulanz):

Ausfüllen des SOKRATES (Version 3), der Subskala „Precontemplation" des VSS-k, des Fragebogens zur Alkoholspezifischen Risikowahrnehmung (FAR) und der deutschen Version der Domain-Specific Risk-Taking Scale (DOSPERT) als Teil der festen Diagnostik- Batterie (bei Standardfragebogenpaket der Ambulanz zu Beginn jeder Behandlung)

Follow-up (nach 3 Monaten):

Ausfüllen des Timeline Follow Back , des SOKRATES (Version 3), der Subskala „Precontemplation" des VSS-k, des Fragebogens zur Alkoholspezifischen Risikowahrnehmung (FAR) und der deutschen Version der Domain-Specific Risk-Taking Scale (DOSPERT).

Im Anschluss: Aufklärung über die Studie.

2. Einverständniserklärung

Information und Einverständniserklärung (Exemplar für Patienten)

Studie: Risikoeinschätzung von Alkohol

Studienleitung: Dr. Michael Odenwald (Universität Konstanz)

Ziel und Zweck der Untersuchung:
Wir untersuchen in dieser Studie Personen[1], die sich in Psychotherapie begeben wollen, bezüglich ihrer Bereitschaft, Ihren Alkoholkonsum zu verändern. Unser Ziel ist es, ein besseres Verständnis für die Risikoeinschätzung von Alkohol zu gewinnen.

Ablauf:
Sie erhalten Fragen zu Ihrer Person, Ihrem Alkohol- und sonstigen Substanzkonsum sowie Ihrer Einschätzung von ihrem Alkoholkonsum und den damit verbundenen Risiken. Sie können die Fragebögen selbständig ausfüllen oder gemeinsam mit einem Mitarbeiter/einer Mitarbeiterin. Sie benötigen dazu heute ca. 10 Minuten. Zu späteren Zeitpunkten werden Sie erneut Fragebögen im Rahmen dieser Studie erhalten.

Risiken
Die Untersuchung beinhaltet keinerlei Risiken und Gefahren. Falls es Ihnen nach der Untersuchung unerwarteterweise schlecht gehen sollte, bitten wir Sie, den Mitarbeiter oder Ihren Therapeuten zu informieren. Diese Information ist wichtig für Ihre weitere Therapie.

Leistung/Kosten
Für die Untersuchung entstehen keine Kosten. Sie erhalten für die Teilnahme kein Geld.

Datenschutz
Die von Ihnen preisgegebenen Informationen unterliegen der Schweigepflicht. Alle Informationen werden streng vertraulich behandelt. Sie werden in verschlossenen Schränken aufbewahrt und nicht an Dritte weitergegeben.
Nur autorisierte Mitarbeiter haben Zugang zu den erhobenen Daten. Die Informationen werden in anonymisierter Form auf gesicherten Computern gespeichert. Die Auswertung der Daten erlaubt keinen Rückschluss auf Ihre Person.

Sonstiges
Die Teilnahme an der Studie ist freiwillig. Sie können sich jederzeit gegen die Teilnahme entscheiden, ohne dass Ihnen ein Nachteil entsteht. Durch Ihre Einwilligung gehen Sie keinerlei Verpflichtungen ein. Sie können die Teilnahme jederzeit und ohne Angabe von Gründen abbrechen, ohne dass Ihnen ein Nachteil entsteht.

Haben Sie Fragen?

---

[1] Zur besseren Lesbarkeit verwenden wir fortan die Bezeichnung „Patient“, „Therapeut“ etc. für beide Geschlechter.

Einverständniserklärung

Ich ____

(Vor- und Zuname des Probanden)

geb. am _____________________________________

nehme freiwillig an der Untersuchung teil. Ich weiß, dass ich die Untersuchung jederzeit abbrechen kann. Ich habe die vorausgehende Erklärung gelesen bzw. sie wurde mir vorgelesen. Ich wurde von den Mitarbeitern über die Studie aufgeklärt und meine Fragen wurden für mich befriedigend und umfassend beantwortet.

Ich weiß, dass Daten über meine Person nicht an Dritte weitergegeben werden dürfen, dass sie im Computer nur anonym gespeichert und verarbeitet werden und dass bei wissenschaftlichen Veröffentlichungen keinerlei Rückschlüsse auf meine Person möglich sind.

Ich weiß, dass die Untersuchung keine Risiken beinhaltet. Falls es mir nach der Untersuchung dennoch schlechter gehen sollte, kann ich mich jederzeit an meinen Therapeuten wenden oder mich mit den Mitarbeitern des Projekts in Verbindung setzen.

Kontakt: _____________________________________ oder michael.odenwald@uni-konstanz.de

Ort, Datum: ____

Unterschrift des Probanden: _________________________________

Unterschrift des Untersuchers: _______________________________

<u>Information und Einverständniserklärung (Exemplar für die Dokumentation)</u>

Studie: Risikoeinschätzung von Alkohol

Studienleitung: Dr. Michael Odenwald (Universität Konstanz)

Ziel und Zweck der Untersuchung:
Wir untersuchen in dieser Studie Personen[2], die sich in Psychotherapie begeben wollen, bezüglich ihrer Bereitschaft, Ihren Alkoholkonsum zu verändern. Unser Ziel ist es, ein besseres Verständnis für die Risikoeinschätzung von Alkohol zu gewinnen.

Ablauf:
Sie erhalten Fragen zu Ihrer Person, Ihrem Alkohol- und sonstigen Substanzkonsum sowie Ihrer Einschätzung von ihrem Alkoholkonsum und damit verbundenen Risiken. Sie können die Fragebögen selbständig ausfüllen oder gemeinsam mit einem Mitarbeiter/einer Mitarbeiterin. Sie benötigen dazu heute ca. 10 Minuten. Zu späteren Zeitpunkten werden Sie erneut Fragebögen im Rahmen dieser Studie erhalten.

Risiken
Die Untersuchung beinhaltet keinerlei Risiken und Gefahren. Falls es Ihnen nach der Untersuchung unerwarteterweise schlecht gehen sollte, bitten wir Sie, den Mitarbeiter oder Ihren Therapeuten zu informieren. Diese Information ist wichtig für Ihre weitere Therapie.

Leistung/Kosten
Für die Untersuchung entstehen keine Kosten. Sie erhalten für die Teilnahme kein Geld.

Datenschutz
Die von Ihnen preisgegebenen Informationen unterliegen der Schweigepflicht. Alle Informationen werden streng vertraulich behandelt. Sie werden in verschlossenen Schränken aufbewahrt und nicht an Dritte weitergegeben.
Nur autorisierte Mitarbeiter haben Zugang zu den erhobenen Daten. Die Informationen werden in anonymisierter Form auf gesicherten Computern gespeichert. Die Auswertung der Daten erlaubt keinen Rückschluss auf Ihre Person.

Sonstiges
Die Teilnahme an der Studie ist freiwillig. Sie können sich jederzeit gegen die Teilnahme entscheiden, ohne dass Ihnen ein Nachteil entsteht. Durch Ihre Einwilligung gehen Sie keinerlei Verpflichtungen ein. Sie können die Teilnahme jederzeit und ohne Angabe von Gründen abbrechen, ohne dass Ihnen ein Nachteil entsteht.

Haben Sie Fragen?

---

[1] Zur besseren Lesbarkeit verwenden wir fortan die Bezeichnung „Patient“, „Therapeut“ etc. für beide Geschlechter.

Einverständniserklärung

Ich ____________________________________
(Vor- und Zuname des Probanden)

geb. am __________________________________

nehme freiwillig an der Untersuchung teil. Ich weiß, dass ich die Untersuchung jederzeit abbrechen kann. Ich habe die vorausgehende Erklärung gelesen bzw. sie wurde mir vorgelesen. Ich wurde von den Mitarbeitern über die Studie aufgeklärt und meine Fragen wurden für mich befriedigend und umfassend beantwortet.

Ich weiß, dass Daten über meine Person nicht an Dritte weitergegeben werden dürfen, dass sie im Computer nur anonym gespeichert und verarbeitet werden und dass bei wissenschaftlichen Veröffentlichungen keinerlei Rückschlüsse auf meine Person möglich sind.

Ich weiß, dass die Untersuchung keine Risiken beinhaltet. Falls es mir nach der Untersuchung dennoch schlechter gehen sollte, kann ich mich jederzeit an meinen Therapeuten wenden oder mich mit den Mitarbeitern des Projekts in Verbindung setzen.

Kontakt: __________________________________ oder michael.odenwald@uni-konstanz.de

Ort, Datum: ____

Unterschrift des Probanden: _______________________________

Unterschrift des Untersuchers: _____________________________

3. Instruktionen für die Teilnehmer

1. Termin (Abklärungsgespräch)

Sehr geehrte Teilnehmerin, sehr geehrter Teilnehmer,

Sie haben heute einen Termin in der Hochschulambulanz wahrgenommen, um abzuklären, ob Sie hier Behandlung für Ihre psychischen Beschwerden erhalten können.

Diese Studie und dieser Fragebogen sollen uns helfen, das therapeutische Angebot zu verbessern.

Wir wollen mit diesem Fragebogen herausfinden, wie Sie sich fühlen würden, wenn hier auch Alkoholkonsum routinemäßig thematisiert werden würde.

Wir bitten Sie freundlich um Ihre Teilnahme. Dr. Michael Odenwald, Leiter der Ambulanz

Alle anderen Termine

Sehr geehrte Teilnehmerin, sehr geehrter Teilnehmer,

Wir bitten Sie, im Rahmen unserer Studie, den Fragebogen zum Umgang mit dem eigenen Trinkverhalten erneut auszufüllen.

Diese Studie und dieser Fragebogen sollen uns helfen, das therapeutische Angebot zu verbessern.

Vielen Dank für Ihre Teilnahme.Dr. Michael Odenwald, Leiter der Ambulanz

Self-report instruments,
Mindset inductions and
intervention manuals
which are part of the original proposal to
the Ethics Commission
will not be reported here.

Universität Konstanz

University of Konstanz - P.O. Box 27 - 78457 Konstanz

Prof. Marcel Leist
Ethics Commission of the University of Konstanz

Dr. Michael Odenwald

Senior Psychologist, Research Ward for Schizophrenia

Head, Psychotherapeutic University Outpatient Clinic for Research & Teaching

Universitätsstraße 10
D-78464 Konstanz
+49 (0) 75 31 / 88 - 4621
Fax +49 (0) 75 31 / 88 - 4838

michael.odenwald@uni-konstanz.de
www.uni-konstanz.de

01.06.2016

Re: Addendum of ethic's proposal, Subproject of the research group "The Dynamics of Risk - Perception and Behavior in the Context of Mental and Physical Health (RISKDYNAMICS)".

Dear Prof. Leist,
Ladies and Gentlemen,

Within the above-mentioned research group I am responsible together with Prof. Brigitte Rockstroh for the subproject P8 "Addiction Risk: The dynamics of risk perception and risk behavior in alcohol dependence". The subproject consists of two independent Work Packages (WP), each WP contains several studies or experiments.

I am mainly responsible for WP1.
Enclosed I send you the procedure, instructions and instruments used in P8 WP1 Study 2.
In this study no drugs are administered or biological interventions are performed. This is about conversation-based interventions and questionnaires.

I ask for the certification of adherence to ethical standards of research.

Yours sincerely

Dr. Michael Odenwald

Title of the study: Preparedness Effects in the Context of Feedback: Variation of Treatment Effects after Mindset Induction

To the background:
Risky alcohol consumption is frequent and harmful. Medical advice and short interventions are efficient ways to reduce hazardous alcohol consumption, but it is unclear what affects the effectiveness of these methods. In this study, we test an experimental paradigm that is designed to make state variables that affect the success of such short interventions investigatable.

To the procedure:
Psychotherapy patients with hazardous alcohol consumption are asked to participate in the study (see appendix for declarations of consent). They are recruited in three psychotherapy outpatient clinics (all in Konstanz): the University Outpatient Clinic for Research and Teaching at the University of Konstanz and the cooperating outpatient clinics of the Tübingen Academy for Behavioural Therapy and the dgvt Training Centre for Psychotherapy Bodensee (apb). According to WHO recommendations, hazardous alcohol consumption is recorded by means of a validated short self-report instrument (Barbor et al., 2001). All patients with hazardous alcohol consumption receive the brief psychological intervention recommended by the World Health Organization (ASSIST-linked Brief Intervention, Humeniuk et al., 2010a; see appendix). The study examines whether this intervention is differently effective depending on the condition of the patients. The state is experimentally induced by a short writing task, in which the participants are asked to think about an interpersonal problem and its solution (Gollwitzer, 2012; see appendix). Randomization includes either the induction of an implemental or a deliberative mindset. The investigator has no knowledge of the type of induced mindset because the subject receives a sealed envelope with one of the two induction tasks with the request to work through the task on his/her own. Implemental Mindset is related to someone being determined to put a decision into action. Deliberative mindset means someone collects and weighs information before making a decision.
Our hypothesis is that depending on the mindset, the willingness varies to accept the advice regarding drinking alcohol.
The brief psychological intervention is carried out by trained psychologists or master students under psychotherapeutic supervision.

On full information of participants:
The participating patients initially do not know that this is a single study; they initially think they are participating in two different surveys that have nothing to do with each other. At the end of the study period, after three months, the patients will be fully informed about the study.

Instruments:
The annex contains the schedule of the study, the information and declarations of agreement and the instruments used, which are briefly described below.

AUDIT:
The Alcohol Use Disorder Identification Test (AUDIT; Barbor et al., 2001) is a screening questionnaire developed by the WHO with 10 items, which are answered with predefined answers. It has a high validity in the identification of hazardous alcohol consumption.

SOCRATES:
The Stages of Change Readiness and Treatment Eagerness Scale (SOCRATES; Miller & Tonigan, 1996) measures therapy motivation in alcohol patients. The German version has a total of 19 simple items, which are answered on a 5-level Likert scale. This instrument is often used in clinical practice. The German version is field-tested and well validated. We use slightly modified versions of the introductory instruction that fit the context better than the original instruction, items and answer scales are not modified.

VSS-k:
The *Veränderungsstadien-Skala* (VSS; Fecht et al., 1998) is the German version of the University of Rhode Island Change Assessments (URICA), which measures change motivation. The German short version VSS-k has 16 items, which are answered on a 5-step Likert scale. The subscale "Precontemplation" measures the state in which there is no insight into the problem and has 4 items. The instrument is well validated and used in many languages. (These 4 items are listed in the Annex as the last 4 items of SKRATES-N).

FAR:
The questionnaire on Alcohol-related Risk Perception (FAR; Klepper et al., under revision) measures various dimensions of the subjective assessment of typical risks arising from the chronic risky consumption of alcohol. It is used here in its short form with 20 items, each of which is answered on a 5-level Likert scale.

DOSPERT
The Domain-Specific Risk-Taking Scale (DOSPERT; Blais & Weber, 2006) is a questionnaire with a total of 60 items that measures the general willingness of a person to show risk behavior, the subjective assessment of risks and the expected benefit of typical risk situations. The DOSPERT is frequently used internationally in research and is regarded as the standard instrument.

TLFB:
The Timeline Follow Back (TLFB; Sobell & Sobell, 1992) is a calendar method that retrospectively measures alcohol consumption by recording standard drinks per calendar day over the period under study. Today it is regarded as the standard procedure for the retrospective recording of alcohol consumption and has been validated many times.

ASSIST:
The Alcohol, Smoking and Substance Involvement Screening Test (ASSIST; Humeniuk et al., 2010b) is a short interview procedure developed by the WHO with 8 questions that can be car-

ried out by trained persons in primary health care. The sum score indicates how risky the reported consumption of different substances is. Here we only use the instrument in connection with alcohol consumption. The questionnaire is the basis of the ASSIST-linked Brief Intervention (Humeniuk et al., 2010a), in which a standardized therapeutic feedback of the interview result is given.

Literature:

Barbor, T.F., Higgings-Biddle, J.C., Saunders, J.B. & Monteiro, M.G. (2001). AUDIT – The alcohol use disorder identification test. guidelines for use in primary Care. 2nd edition. Geneva: World Health Organization.

Blais, A-R. and E.U. Weber (2006). A domain-specific risk-taking (DOSPERT) scale for sdult populations. *Judgment and Decision Making,* 1, 33-47.

Fecht, J., Heidenreich, T., Hoyer, J., Lauterbach, W., & Schneider, R. (1998). Stages of change in inpatient alcohol withdrawal treatment: diagnostic problems. *Behavioural Therapy and Psychosocial Practice, 30*, 403-419.

Gollwitzer, P.M. (2012). Mindset theory of action phases. In P. Van Lange, A.W. Kruglanski & E.T. Higgins (Eds.), *Handbook of Theories of Social Psychology* (pp. 526-545). London: Sage Publications.

Humeniuk, R., Henry-Edwards, S., Ali, R., Poznyak, V., & Monteiro, M. G. (2010a). The ASSIST-linked brief intervention for hazardous and harmful substance use: a manual for use in primary care. Geneva: World Health Organization.

Humeniuk, R., Henry-Edwards, S., Ali, R., Poznyak, V., & Monteiro, M. G. (2010b). The Alcohol, Smoking and Substance Involvement Screening Test (ASSIST): manual for use in primary care. Geneva: World Health Organization.

Klepper, S.I., Odenwald, M., Rockstroh, B. (in revision). Risk perception and alcohol dependence. *Search*.

Miller, W. R., & Tonigan, J. S. (1996). Assessing drinkers' motivation for change: The Stages of Change Readiness and Treatment Eagerness Scale (SOCRATES). *Psychology of Addictive Behaviors* 10, 81-89.

Sobell, L. C., & Sobell, M. B. (1992). Timeline follow-back: A technique for assessing selfreported alcohol consumption. In R.Z. Litten & J. Allen (Eds.). *Measuring alcohol consumption: Psychosocial and Biological Methods* (pp. 41-72). New Jersey: Humana Press..

Annex

1. Plan of the procedures of the study (schedule)
2. Declaration of consent
3. Instructions for participants
4. Self-report instruments
5. Mindset-Induction
5.1 Deliberative Mindset
5.2 Implemental Mindset
6. ASSIST and ASSIST-linked Brief Intervention

1. Plan of the procedures of the study (schedule)

Schedule of the study

1st appointment (initial meeting for clarification and information):

A 30-minute consultation with an experienced therapist takes place before psychotherapy begins in the participating outpatient clinics.

At the end of this consultation when suitability for psychotherapy has ben determined and the patients has decided to start psychotherapy at the outpatient clinic, he/she will be informed about the study and asked whether he/ she agrees to participate in the study.

Thereupon: Filling in the consent form, the AUDIT and the SOCRATES (version 1), the subscale “Precontemplation” oft he VSS-k, the questionnaire on Alcohol-specific Risk Perception (FAR) and the German version oft he Domain-Specific Risk-Taking Scale (DOSPERT).

If an AUDIT cut-off value of 5 is reached, patients will be included in the intervention part of the study and further study appointments will be arranged in addition to the appointments for ongoing psychotherapy.

2nd appointment:

(1) Mindset induction (50% oft he participants receive the instruction „weighing“, the other 50% the instructions „making plans“)
(2) Completing the Timeline Follow Back (over the last four weeks)
(3) Filling in the SOCRATES (version 2) and the subscale “Precontemplation” of the VSS-k
(4) Conducting the ASSIST interview
(5) Implementation of the ASSIST-linked Brief Intervention

3rd appointment (standard diagnostics o the outpatient clinic):

Completion oft he SOCRSTES (version 3), the subscale “Precontemplation” of the VSS-k, the questionnaire on Alcohol-specific Risk Perception (FAR) and the German version of the Domain-Specific Risk-Taking Scale (DOSPERT) as part of the standard diagnostic battery (the standard questionnaire package of the outpatient clinic is filled in at the beginning of each treatment).

Follow-up (after 3 months):

Filling in the Timeline Follow Back, the SOCRATES (version 3), the subscale “Precontemplation” of the VSS-k, the questionnaire on Alcohol-specific Risk Perception (FAR) and the German version of the Domain-Specific Risk-Taking Scale (DOSPERT).

Subsequently: Providing full information about the study.

2. Declaration of consent

<u>Information and declaration of consent (copy for patient)</u>

Study: Risk Assessment of Alcohol

Head of Study: Dr. Michael Odenwald (University of Konstanz)

Aim and purpose of the investigation:

In this study, we are investigating individuals[1] who want to go into psychotherapy with regard to their willingness to change their alcohol consumption. Our aim is to gain a better understanding of alcohol-related risk perception.

Procedure:

You will receive questions about yourself, your consumption of alcohol and other substances as well as your assessment of your alcohol consumption and the associated risks. You can fill out the questionnaires yourself or together with a member of the research team. You will need about 10 minutes today. At a later date, you will receive new questionnaires as part of this study.

Risks:

The investigation does not include any risks or dangers. If you unexpectedly feel unwell after the examination, please inform the staff member or your therapist. This information is important for your further therapy.

Services/Costs:

There are no costs for the investigation. You will not receive any money for participation.

Data protection:

The information provided by you is subject to confidentiality. All information will be kept strictly confidential. They are stored in locked cabinets and are not passed on to third parties.
Only authorized employees have access to the collected data. The information is stored anonymously on secure computers. The analysis of the data does not allow any conclusions to be drawn about your person.

Other:

Participation in the study is voluntary. You can decide not to participate at any time without being disadvantaged. By your consent you do not enter into any obligations. You may cancel your participation at any time and without giving any reason, without incurring any disadvantage.

Do you have any questions?

---

[1] For better readability, a gender-neutral term such as "patient" or "therapist" will be used for both sexes.

Consent form

I'm ______________________________________
(First name and surname of the participant)

born on ____________________________________

volunteer to participate in the investigation. I know I can abort the investigation at any time. I have read the previous statement or it has been read to me. I was informed about the study by the staff and my questions were answered satisfactorily and comprehensively.

I know that data about my person will not be passed on to third parties, that they are only stored and processed anonymously in the computer and that no conclusions about my person are possible in the case of scientific publications.

I know the investigation doesn't involve any risks. If I still feel worse after the examination, I can contact my therapist or the project staff at any time.

Contact: ___________________________________or michael.odenwald@uni-konstanz.de

Place, date: ____

Signature of the participants: ________________________________

Signature of the investigator: ______________________________

Information and declaration of consent (copy for project documentation)

Study: Risk Assessment of Alcohol

Head of Study: Dr. Michael Odenwald (University of Konstanz)

Aim and purpose of the investigation:

In this study, we are investigating individuals[2] who want to go into psychotherapy with regard to their willingness to change their alcohol consumption. Our aim is to gain a better understanding of alcohol-related risk perception.

Procedure:

You will receive questions about yourself, your consumption of alcohol and other substances as well as your assessment of your alcohol consumption and the associated risks. You can fill out the questionnaires yourself or together with a member of the research team. You will need about 10 minutes today. At a later date, you will receive new questionnaires as part of this study.

Risks:

The investigation does not include any risks or dangers. If you unexpectedly feel unwell after the examination, please inform the staff member or your therapist. This information is important for your further therapy.

Services/Costs:

There are no costs for the investigation. You will not receive any money for participation.

Data protection:

The information provided by you is subject to confidentiality. All information will be kept strictly confidential. They are stored in locked cabinets and are not passed on to third parties.
Only authorized employees have access to the collected data. The information is stored anonymously on secure computers. The analysis of the data does not allow any conclusions to be drawn about your person.

Other:

Participation in the study is voluntary. You can decide not to participate at any time without being disadvantaged. By your consent you do not enter into any obligations. You may cancel your participation at any time and without giving any reason, without incurring any disadvantage.

Do you have any questions?

[2] For better readability, a gender-neutral term such as “patient” or “therapist” will be used for both sexes.

Consent form

I'm ___________________________________
(First name and surname of the participant)

born on _________________________________

volunteer to participate in the investigation. I know I can abort the investigation at any time. I have read the previous statement or it has been read to me. I was informed about the study by the staff and my questions were answered satisfactorily and comprehensively.

I know that data about my person will not be passed on to third parties, that they are only stored and processed anonymously in the computer and that no conclusions about my person are possible in the case of scientific publications.

I know the investigation doesn't involve any risks. If I still feel worse after the examination, I can contact my therapist or the project staff at any time.

Contact: _________________________________or michael.odenwald@uni-konstanz.de

Place, date: ____

Signature of the participants: ______________________________

Signature of the investigator: ____________________________

3. Instructions for participants

1st appointment (initial meeting for clarification and information):

Dear participant,

Today you have participated in an appointment of the University outpatient clinic to clarify whether you will receive treatment for your psychological complaints.

This study and this questionnaire are thought to help us to further develop and improve the range of therapeutic services we offer.

With this questionnaire we want to find out, how you feel if alcohol consumption is routinely made to a topic in psychotherapy.

We kindly ask for your participation.

Dr. Michael Odenwald, Head of the outpatient clinic

All other appointments

Dear participant,

We kindly ask you to again fill in the questionnaire on how you see your own drinking behavior.

This study and this questionnaire are thought to help us to improve the range of therapeutic services.

Thank you very much for your participation.

Michael Odenwald, Head of the outpatient clinic

Self-report instruments,
Mindset inductions and
intervention manuals
which are part of the original proposal to
the Ethics Commission
will not be reported here.

Universität Konstanz

Universität Konstanz · Postfach 27 · 78457 Konstanz

Herrn Prof. Marcel Leist
Ethikkommission der Universität Konstanz

Dr. Michael Odenwald

Leitender Psychologe, Forschungsstation für Schizophrenie

Leiter, Psychotherapeutische Hochschulambulanz für Forschung & Lehre

Universitätsstraße 10
D-78464 Konstanz
+49 (0) 75 31 / 88 - 4621
Fax +49 (0) 75 31 / 88 - 4838

michael.odenwald@uni-konstanz.de
www.uni-konstanz.de

23.10.2017

Betr.: Nachtrag Ethikantrag Teilprojekt der Forschergruppe „The Dynamics of Risk – Perception and Behavior in the Context of Mental and Physical health (RISKDYNAMICS)“

Sehr geehrter Herr Prof. Leist,
sehr geehrte Damen und Herren,

im Rahmen der o.g. Forschergruppe RISKDYNAMICS (Sprecher Britta Renner und Harald Schupp) bin ich zusammen mit Prof. Brigitte Rockstroh für das Teilprojekt P8 „Addiction Risk: Die Dynamik von Risikowahrnehmung und Risikoverhalten bei Alkoholabhängigkeit“ verantwortlich. Das Teilprojekt besteht aus zwei unabhängigen Work Packages (WP), jedes WP beinhaltet mehrere Studien bzw. Experimente. Für WP1 bin ich hauptverantwortlich.

Ich beantrage hiermit die ethische Prüfung von Änderungen einer zuvor schon von Ihnen geprüften und bereits genehmigten Studie unseres Teilprojektes der o.g. Forschergruppe.

Die Änderungen betreffen **Studie 2 „Preparedness Effects in the Context of Feedback: Variation of Treatment Effects after Mindset Induction“.** Angesichts von Schwierigkeiten bei der Durchführung der Studie an PatientInnen in den beiden beteiligten Ambulanzen, möchten wir die Studie nun im WS 17/18 an einer studentischen Stichprobe durchführen. Dazu wird der Ablauf geringfügig verändert, um den Rahmenbedingungen Rechnung zu tragen.

Ich bitte um die Prüfung und Bescheinigung der ethischen Unbedenklichkeit der Änderungen der Studie.

Mit freundlichen Grüßen

Michael Odenwald

Dr. Michael Odenwald

Betr.: Nachtrag Ethikantrag Teilprojekt der Forschergruppe „The Dynamics of Risk – Perception and Behavior in the Context of Mental and Physical health (RISKDYNAMICS)"

 23.10.2017

Anhänge:

1. Bisherige Bescheinigungen der Ethikkommission

2. Beschreibung der Änderungen

3. Informationsblatt und Einverständniserklärung

4. Zusätzliche Items

Universität Konstanz

Universität Konstanz · Fach 224 · 78457 Konstanz

Herr
Dr. Michael Odenwald
FB Psychologie
Fach 27
im Hause

Sabine Schieß
Ethikkommission

Universitätsstraße 10
D-78464 Konstanz
+49 7531 88-5264
Fax +49 7531 88-3727

sabine.schiess@uni-konstanz.de
www.uni-konstanz.de

19.07.2016

BESTÄTIGUNG 21/2016

Die Universität Konstanz stellt fest, dass gegen die Durchführung des

P8 WP 1 Studie 2 im Rahmen der Forschergruppe RISKDYNAMICS

keine Bedenken bestehen.

Grundlagen der Beurteilung sind:

1. Die Deklaration von Helsinki in der vom Weltärztebund beschlossenen aktuellen Fassung.
2. Ihr Antrag an die Ethik-Kommission mit dem oben genannten Thema.

Die Sekretärin der Ethik-Kommission

Sabine Schieß

Anhang 2:

Studie „Preparedness Effects in the Context of Feedback: Variation of Treatment Effects after Mindset Induction“

Aktueller Stand der Studie und ausführliche Beschreibung der Änderungen

1. Ursprünglich geplante Studie:

Ziel der Studie ist die Weiterentwicklung von existierenden Kurzzeitinterventionen zur Verringerung von riskantem Alkoholkonsum. Hierzu sollte etabliertes Wissen aus einem psychologischen Grundlagenfachgebiet (Sozialpsychologie) sowie die Erkenntnisse der Risikoforschung in die klinische Interventionsforschung aufgenommen werden. In dieser Studie sollten PsychotherapiepatientInnen, die neben der psychischen Probleme riskanten Alkoholkonsum aufweisen, involviert werden. Patientinnen werden auf Basis des Screeningfragebogens (AUDIT; Barbor et al., 2001) für die Studienteilnahme ausgewählt (Wert >4 deutet auf riskanten Alkoholkonsum hin). Alle Studienteilnehmer erhalten die von der WHO empfohlene und gut validierte ASSIST-linked Brief Intervention (Humeniuk et al., 2010), welche die Patienten motiviert, ihren Alkoholkonsum zu verringern. In dieser Studie werden durch psychologische Schreibaufgaben (Entscheidungsaufgaben) zwei unterschiedliche „Mindsets“ experimentell induziert; deren Wirkung untersuchen wir auf die Wirkung der nachfolgenden Kurzintervention zur Alkoholreduktion. Wir erwarten eine Verbesserung der Wirkung der Intervention, wenn Patienten im deliberative Mindset sind.

Das Design enthält drei Messzeitpunkte:

1. Baseline
2. Experimentelle Sitzung mit Mindset-Induktion und Kurzintervention
3. Follow-up nach 3 Monaten

Geplant war, diese Studie an 100 Patienten der Hochschulambulanz für Forschung und Lehre (Universität Konstanz) und kooperierenden Ambulanzen durchzuführen.

2. Ausführliche Beschreibung der Änderungen:

Aktuell haben sich aufgrund der durch Wegnahme von Räumen zugespitzten Raumnot der Hochschulambulanz und der organisatorischen Probleme seitens der Partnerambulanzen zu wenige Patienten in die Studie einbinden lassen. Wir erwarten, dass wir lediglich 40 statt 100 PatientInnen in die Studie aufnehmen können.

Das geplante Vorhaben soll nun in geänderter Form durchgeführt werden, um die theoretischen Annahmen an einer weiteren und ausreichend großen Stichprobe überprüfen zu können.

Wir planen daher dieselbe Studie an einer studentischen Population an der Universität Konstanz durchzuführen.

Folgende Modifikationen des Designs und des Ablaufs sind vorgesehen:

1. Durchführung der Studie an einer studentischen Stichprobe zusätzlich zur Patientenstichprobe.
2. Aus ökonomischen Gründen erfolgt die Rekrutierung der studentischen Teilnehmer und Terminvergabe mittels der online-Versuchsplattform SONA des Fachbereichs Psychologie:
   https://uni-konstanz.sona-systems.com/Default.aspx?ReturnUrl=%2f
   SONA ist eine professionelle online-Versuchsplattform, für die der Fachbereich Psychologie eine Lizenz besitzt und die die Anonymität von Versuchspersonen wahrt. Studierende tragen sich in SONA ein, um die erforderlichen Versuchspersonen-

Stunden zu sammeln. Üblicherweise nehmen Studierende an einer Vielzahl von Experimenten und Studien teil, die über SONA organisiert werden. Im Anhang die Informationen und Einverständniserklärung bei der Anmeldung zu SONA.

3. In SONA wird der Baseline- und Follow-up-Fragebogen mittels der Online-Plattform Unipark stattfinden. SONA ist durch ein Interface an Unipark angebunden. Dadurch können die studentischen Teilnehmer diese Fragebögen an ihrem eigenen PC, unabhängig vom Ort, zu einer ihnen passenden Zeit durchführen. In der ursprünglichen Studie mit Patienten wurden die Fragebögen in der Paper-Pencil-Version in der Ambulanz ausgefüllt. Bevor die Teilnehmer die Baseline-Erhebung auf Unipark beginnen, lesen sie die Information und Einverständniserklärung. Durch Ankreuzen eines Kästchens geben Sie ihr Einverständnis. Siehe Anhang.
4. Um die Daten aus den drei Sitzungen einander zuordnen zu können und um die Anonymität zu bewahren, wird in jeder Sitzung jeweils unabhängig voneinander ein Versuchspersonen-Code generiert. Der Versuchspersonen-Code wird vom Teilnehmer selbst erstellt durch die Angabe folgender 6-stelliger Buchstaben-Ziffern-Folge:
    a. Stelle 1 und 2: erste zwei Buchstaben des Vornamens der Mutter
    b. Stelle 3 und 4: Ziffern des Tags des eigenen Geburtstags
    c. Stelle 5 und 6: erste zwei Buchstaben des eigenen Geburtsorts
5. Wie im ursprünglichen Studiendesign wird den Versuchspersonen zu Beginn der Studie verschwiegen, dass nur diejenigen Studierenden, die einen riskanten Alkoholkonsum angeben, zu den Terminen 2 und 3 eingeladen werden; stattdessen wird die Information gegeben, dass ein Teil der Teilnehmer zufällig zu weiteren Untersuchungen ausgewählt wird. Ferner wird bei der experimentellen Sitzung 2 ähnlich wie bisher die Information gegeben, es handle sich bei Bearbeitung der Entscheidungsaufgabe (Mindsetinduktion) und Kurzintervention um mehrere nicht zusammenhängende Studien. Alle Studienteilnehmer (auch diejenigen, die nur an Termin 1 teilgenommen hatten) werden nun nach dem Abschluss aller Follow-up-Messungen über die SONA-Plattform per Email über das wirkliche Design der Studie aufgeklärt; in der ursprünglichen Version sollte den Patienten diese Information in einem persönlichen Gespräch gegeben werden.
6. Zusätzlicher Einschluss des Time-Line-Follow-Back Fragebogens (Sobell & Sobell, 1992; Kurzversion) in die Baseline Messung. Bislang kam dieser Fragebogen nur in der experimentellen Sitzung und der Follow-up-Messung zur Anwendung. Insgesamt wird dieser Fragebogen daher drei mal statt zwei mal angewendet.
7. Zusätzlich sollen 6 Items zur Messung von Risikoverhalten aus dem Sozioökonomischen Panel (SOEP; Josef et al., 2016) mit eingeschlossen werden. Diese Items gelten als gut validiert und werden zur ökonomischen Erfassung des Risikoverhaltens in vielen Studien eingesetzt. Siehe diese Items und Information zum SOEP im Anhang.
8. Entschädigung der studentischen Versuchspersonen für die Teilnahme durch Versuchspersonenstunden oder Versuchspersonengelder (10€/h).
9. Follow-up Messung findet nach 4 Wochen statt nach 3 Monaten, da die Studie im Rahmen eines Semester-Zyklus abgeschlossen werden muss.

Literatur:

Barbor, T.F., Higgings-Biddle, J.C., Saunders, J.B. & Monteiro, M.G. (2001). AUDIT – The alcohol use disorder identification test. guidelines for use in primary Care. 2nd edition. Geneva: World Health Organization.

Gollwitzer, P.M. (2012). Mindset theory of action phases. In P. Van Lange, A.W. Kruglanski & E.T. Higgins (Eds.), *Handbook of Theories of Social Psychology* (pp. 526-545). London: Sage Publications.

Humeniuk, R., Henry-Edwards, S., Ali, R., Poznyak, V., & Monteiro, M. G. (2010). The ASSIST-linked brief intervention for hazardous and harmful substance use: a manual for use in primary care. Geneva: World Health Organization.

Josef, A.K., Richter, D., Samanez-Larkin, G.R., Wagner, G.G., Hertwig, R., Mata, R. (2016). Stability and change in risk-taking across the adult life span. Journal of Personality and Social Psychology, Vol. 111(3), 430-450.

Sobell, L. C., & Sobell, M. B. (1992). Timeline follow-back: A technique for assessing selfreported alcohol consumption. In R.Z. Litten & J. Allen (Eds.). *Measuring alcohol consumption: Psychosocial and Biological Methods* (pp. 41-72). New Jersey: Humana Press

Anhang 3.1

Einverständniserklärung bei Anmeldung der Teilnehmer auf der online-Versuchsplattform SONA

Important Human Subject and Privacy Policy Notice
Sowohl Versuchsperson, als auch Versuchsleitung müssen folgenden Richtlinien zur Versuchsdurchführung mit Menschen und zum Datenschutz zustimmen, bevor sie Gebrauch des Study Pools der Universität Konstanz machen dürfen. Sowohl Versuchsleitung als auch Versuchsperson verpflichten sich darüber hinaus zu einem respektvollen gegenseitigen Umgang. Falls Fragen zu Richtlinien, Rechten und Verantwortungen bestehen, wenden Sie sich bitte an: *sona@uni-konstanz.de*.

Besonderheiten psychologischer Versuche
Psychologische Forschung ist auf die Teilnahme von Menschen als Versuchspersonen angewiesen. Psychologen sind sich der Besonderheit der Rollenbeziehung zwischen Versuchsleitung und Versuchspersonen und der daraus resultierenden Verantwortung bewusst. Sie stellen sicher, dass durch die Forschung Würde und Integrität der teilnehmenden Personen nicht beeinträchtigt werden. Sie treffen alle geeigneten Maßnahmen, Sicherheit und Wohl der an der Forschung teilnehmenden Personen zu gewährleisten und versuchen, Risiken auszuschließen.

Datenschutz
Die im System erfassten Daten dienen ausschließlich der Organisation von wissenschaftlichen Experimenten. Die Daten werden nicht an Dritte weitergegeben und nur zu folgenden Zwecken benutzt:
- Einladung von Personen zu Experimenten
- Durchführung wissenschaftlich motivierter Auswahl von Personen zur Teilnahme an bestimmten Experimenten
- Überprüfung des Erscheinens bzw. Nicht-Erscheinens angemeldeter Personen bei Experimenten

Es existiert außerdem keine Verknüpfung zwischen den in den jeweiligen Experimenten erhobenen Daten und den Daten im Study Pool (mit Ausnahme der Daten der Vorerhebung, die der Versuchsleitung zur Verfügung stehen).
Informationen über die Studienteilnahmen von Versuchspersonen sind nur für die jeweiligen Versuchsleitungen sowie den Administratoren von SONA zugänglich. Besteht dennoch Verdacht auf Missbrauch der Privatsphäre, wenden Sie sich bitte an sona@uni-konstanz.de.

Regeln für den gegenseitigen Umgang (Rules of Conduct)
Versuchsleitung als auch Versuchspersonen verpflichten sich zu einem respektvollen gegenseitigen Umgang. Äußerungen, die die Persönlichkeitsrechte von Versuchspersonen oder Versuchsleitung verletzen, sind zu unterlassen. Dazu gehören insbesondere sexuelle Anspielungen und sexistische oder rassistische Beleidigungen. Ein Verstoß gegen diese Regeln des gegenseitigen Umgangs hat den Ausschluss aus der Datenbank zur Folge. Wenn Sie der Meinung sind, dass jemand gegen diese grundsätzlichen Regeln verstoßen hat, schicken Sie uns bitte eine E-Mail an sona@uni-konstanz.de.

Nutzungsrechte
- Die Teilnahme an Versuchen ist vollkommen freiwillig und kann jederzeit ohne Angabe von Gründen und ohne etwaige Nachteile abgebrochen werden. Außerdem können erhobene Daten auf den Wunsch des Teilnehmers gelöscht werden.
- Solange nicht vom Recht auf Abbruch Gebrauch gemacht wird, müssen die Versuchspersonen den Anweisungen der Versuchsleitung Folge leisten. Ein Nichtbeachten der Anweisungen kann zum Ausschluss von Experiment und Bezahlung führen.
- Sollte man sich dazu entscheiden die Fragen etwa zu Alter und Geschlecht in der Vorerhebung zu beantworten, dann müssen die gemachten Angaben auch der Wahrheit entsprechen. Ansonsten kann die willentliche Täuschung zum Ausschluss aus der Datenbank füh-

ren.

Sonstiges
Die Person, die sich für einen Versuch anmeldet, muss auch die Person sein, die zum Versuch erscheint. Anmeldungen für andere Personen sind strengstens untersagt. In manchen Fällen werden Versuchsteilnehmer vom Versuchsleiter zu einer gültigen Identifikation aufgefordert.

Anhang 3.2

Einverständniserklärung der Studie in Unipark:

Information und Einverständniserklärung
Studie: Risikoeinschätzung von Alkohol ***Studienleitung***: Dr. Michael Odenwald (Universität Konstanz)
Ziel und Zweck der Untersuchung: Wir untersuchen in dieser Studie Personen bezüglich ihrer Bereitschaft, ihren Alkoholkonsum zu verändern. Unser Ziel ist es, ein besseres Verständnis für die Risikoeinschätzung von Alkohol zu gewinnen.
Ablauf: Sie erhalten Fragen zu Ihrer Person, Ihrem Alkohol- und sonstigen Substanzkonsum sowie Ihrer Einschätzung von Ihrem Alkoholkonsum und den damit verbundenen Risiken. Sie benötigen dazu heute ca. 15-20 Minuten. Einen Teil der Teilnehmenden laden wir zu einem späteren Zeitpunkt zu weiteren Terminen ein.
Risiken: Die Untersuchung beinhaltet keinerlei Risiken und Gefahren.
Leistung/Kosten: Für die Untersuchung entstehen Ihnen keine Kosten. Sie erhalten für die Teilnahme entweder eine halbe Versuchspersonenstunde oder 5€. Sie können am Ende der Studie zwischen den beiden Vergütungen wählen.
Datenschutz: Die von Ihnen preisgegebenen Informationen unterliegen der Schweigepflicht. Alle Informationen werden streng vertraulich behandelt. Sie werden in verschlossenen Schränken aufbewahrt und nicht an Dritte weitergegeben. Nur autorisierte Mitarbeiterinnen und Mitarbeiter haben Zugang zu den erhobenen Daten. Die Informationen werden in anonymisierter Form auf gesicherten Computern gespeichert. Die Auswertung der Daten erlaubt keinen Rückschluss auf Ihre Person.
Sonstiges: Die Teilnahme an der Studie ist freiwillig. Sie können sich jederzeit gegen die Teilnahme entscheiden, ohne dass Ihnen ein Nachteil entsteht. Durch Ihre Einwilligung gehen Sie keinerlei Verpflichtungen ein. Sie können die Teilnahme jederzeit und ohne Angabe von Gründen abbrechen, ohne dass Ihnen ein Nachteil entsteht.
Haben Sie Fragen? Dann wenden Sie sich an michael.odenwald@uni-konstanz.de.

Mit Klick auf "Ich möchte teilnehmen!" stimmen Sie dem Folgenden zu: Ich nehme freiwillig an der Untersuchung teil. Ich weiß, dass ich die Untersuchung jederzeit abbrechen kann. Ich habe die vorausgehende Erklärung gelesen. Ich weiß, dass Daten über meine Person nicht an Dritte weitergegeben werden dürfen, dass sie im Computer nur anonym gespeichert und verarbeitet werden und dass bei wissenschaftlichen Veröffentlichungen keinerlei Rückschlüsse auf meine Person möglich sind. Ich weiß, dass die Untersuchung keine Risiken beinhaltet.
☐ Ich möchte teilnehmen!
☐ Nein, ich möchte nicht teilnehmen.

Anhang 4

Zusätzliche Items aus dem Sozioökonomischen Panel

Man kann sich in verschiedenen Bereichen unterschiedlich verhalten. Wie würden Sie Ihre Risikobereitschaft in Bezug auf die folgenden Bereiche einschätzen?
Wie risikobereit sind Sie...

	gar nicht risikobereit									sehr risikobereit
...beim Vertrauen in fremde Menschen?	○	○	○	○	○	○	○	○	○	○
...beim Autofahren?	○	○	○	○	○	○	○	○	○	○
...bei Freizeit und Sport?	○	○	○	○	○	○	○	○	○	○
...bei Geldanlagen?	○	○	○	○	○	○	○	○	○	○
...bei Ihrer Gesundheit?	○	○	○	○	○	○	○	○	○	○
...bei Ihrer beruflichen Karriere?	○	○	○	○	○	○	○	○	○	○

Information von der Webseite des Max-Planck-Instituts für Bildungsforschung:
https://www.mpib-berlin.mpg.de/de/presse/2016/01/risikobereitschaft-veraendert-sich-in-jungen-jahren-und-im-hoeheren-alter-am-staerksten

Das Sozio-oekonomische Panel (SOEP) ist eine repräsentative Multi-Kohortenbefragung, die seit 1984 durchgeführt wird. Für die unter dem Dach der Leibniz-Gemeinschaft am DIW Berlin geleiteten Studie werden zur Zeit jedes Jahr in Deutschland etwa 30.000 Personen in fast 15.000 Haushalten von TNS Infratest Sozialforschung (München) in persönlichen Interviews befragt. Beispielsweise geben diese Auskunft zu persönlichen Einstellungen und politischen Ansichten, Einkommen, Erwerbstätigkeit, Bildung oder Gesundheit. Da jedes Jahr dieselben Personen befragt werden, können langfristige psychologische, wirtschaftliche, gesellschaftliche und soziale Entwicklungen aufgezeigt werden.

University of Konstanz - P.O. Box 27 - 78457 Konstanz

Prof. Marcel Leist
Ethics Commission of the University of Konstanz

Dr. Michael Odenwald

Senior Psychologist, Research Ward for Schizophrenia

Head, Psychotherapeutic University Outpatient Clinic for Research & Teaching

Universitätsstraße 10
D-78464 Konstanz
+49 (0) 75 31 / 88 - 4621
Fax +49 (0) 75 31 / 88 - 4838

michael.odenwald@uni-konstanz.de
www.uni-konstanz.de

23.10.2017

Re.: Amendment Subproject of the research group "The Dynamics of Risk - Perception and Behavior in the Context of Mental and Physical Health (RISKDYNAMICS)".

Dear Prof. Leist,
Ladies and Gentlemen,

In the framework of the above mentioned Research Group RISKDYNAMICS (spokespersons Britta Renner and Harald Schupp) I am responsible together with Prof. Brigitte Rockstroh for the subproject P8 "Addiction Risk: The dynamics of risk perception and risk behavior in alcohol dependence". The subproject consists of two independent Work Packages (WP), each WP contains several studies or experiments. I am mainly responsible for WP1.

I hereby apply for the ethical review of changes to a study of our subproject of the above-mentioned research group that you have already reviewed and approved.

The changes concern **Study 2 "Preparedness Effects in the Context of Feedback: Variation of Treatment Effects after Mindset Induction".** In view of the difficulties in conducting the study on patients in the two outpatient departments involved, we would now like to conduct the study in winter term 2017/18 on a student sample. To this end, the process is slightly modified to take account of the framework conditions.

I ask for the examination and certification of the adherence to the ethical standards of research of the amended study.

Yours sincerely

Dr. Michael Odenwald

Universität Konstanz

Re.: Amendment Subproject of the research group "The Dynamics of Risk - Perception and Behavior in the Context of Mental and Physical Health (RISKDYNAMICS)".

23.10.2017

Attachments:

Previous certificates of the ethics committee

Description of changes

Amended Information Sheet and Declaration of Consent

Additional Items

Mr.
Dr. Michael Odenwald
Department of Psychology
Box 27
In-house

Sabine Schieß
Ethics Commission
Universitätsstr. 10
D-78464 Konstanz
+49 7531 88 5264
Fax +49 7531 88 3727
Sabine.schieß@uni-konstanz.de
www.uni-konstanz.de

07/19/2016

Certification 21/2016

Page 1/1

The University of Konstanz states that there are no concerns against the implementation of
P8 WP1 Study 2 in the context oft he Research Group RISKDYNAMICS.

This statement s based on

1. the most recent version of the Declaration of Helsinki adopted by the World Medical Association
2. Your application to the Ethics Commission related to the above mentioned topic

Secretary oft he Ethis Commission

Sabine Schieß

Annex 2:

Study „Preparedness Effects in the Context of Feedback: Variation of Treatment Effects after Mindset Induction".

Current status of the study implementation and detailed description of amendments

1. Originally planned study:

The aim of the study is to further develop existing short-term interventions to reduce hazardous alcohol consumption. For this purpose, established knowledge from a basic psychological field (social psychology) and the findings of risk research should be incorporated into clinical intervention research. In this study it was originally planned to involve psychotherapy patients who, in addition to psychological problems, have risky alcohol consumption. Patients are selected to participate in the study based on a screening questionnaire (AUDIT; Barbor et al., 2001; critical value >4, indicates risky alcohol consumption). All study participants receive the WHO recommended and well validated ASSIST-linked brief intervention (Humeniuk et al., 2010), which motivates patients to reduce their alcohol consumption. In this study, two different "mindsets" are experimentally induced by psychological writing tasks (decision tasks); we investigate their effect on the effect of the subsequent short intervention on alcohol reduction. We expect an improvement in the effect of the intervention when patients are in the deliberative mindset.

The design contains three measurement points:

1. baseline
2. Experimental session with mindset induction and brief intervention
3. Follow-up after 3 months

The plan was to carry out this study on 100 patients of the University Outpatient Clinic for Research and Teaching (University of Konstanz) and cooperating outpatient clinics.

2. Detailed description of the changes:

Currently, due to the lack of space in the university outpatient department due to the removal of rooms and the organizational problems on the part of the partner outpatient departments, too few patients could have been included in the study. We expect to be able to admit only 40 instead of 100 patients to the study.

The planned project is now to be carried out in a modified form in order to be able to verify the theoretical assumptions on a further and sufficiently large sample.

We therefore plan to carry out the same study on a student population at the University of Konstanz.

The following modifications of the design and the procedure are planned:

1. Conduct the study on a student sample in addition to the patient sample.
2. For economic reasons, the recruitment of student participants and the allocation of appointments is carried out using the online test platform SONA of the Department of Psychology:
   https://uni-konstanz.sona-systems.com/Default.aspx?ReturnUrl=%2f
   SONA is a professional online testing platform for which the Department of Psychology is licensed and which preserves the anonymity of test subjects. Students register in SONA in order to collect the necessary test person hours. Usually, students participate in a variety of experiments and studies organized by SONA. In the appendix please find the information and declaration of consent with the registration to SONA.
3. In SONA, the baseline and follow-up questionnaire will take place via the Unipark online platform. SONA is connected to Unipark via an interface. This allows the student partic-

ipants to complete these questionnaires on their own PC, regardless of location, at a time convenient to them. In the original study with patients, paper-pencil versions of the questionnaires were completed in the outpatient department. Before the participants begin the baseline survey at Unipark, they are required to read the information and declaration of consent. By ticking a box they give their consent. See Annex.

4. In order to assign the data from the three separate sessions to each other and to preserve anonymity, an ID-code is generated in each session. The test ID-code is created by the participant himself by generating the following sequence of six letters and digits:
    a. Digits 1 and 2: first two letters of the first name of the mother
    b. Digit 3 and 4: digits of the day of the own birthday
    c. Positions 5 and 6: first two letters of own place of birth
5. As in the original study design, at the beginning of the study the test subjects were not informed that only those students who reported risky alcohol consumption were invited to appointments 2 and 3; instead, the information was given that some of the participants were randomly selected for further examinations. Furthermore, in experimental session 2 the information is given, similar to the previous session, that the decision task (mindset induction) and the short intervention are independent studies that are not connected. All study participants (including those who only attended appointment 1) will now be informed by email about the actual design of the study after completion of all follow-up measurements via the SONA platform; in the original version of the study this information was to be given to the patients in a personal conversation.
6. Additional inclusion of the Time-Line-Follow-Back Questionnaire (Sobell & Sobell, 1992; short version) in the baseline assessment. So far, this questionnaire has only been used in the experimental session and follow-up measurement. This questionnaire is therefore used three times instead of twice.
7. In addition, 6 items for measuring risk behavior from the Socio-Economic Panel (SOEP; Josef et al., 2016) are to be included. These items are considered to be well validated and are used in many studies for the economic assessment of risk behavior. See these items and SOEP information in the appendix.
8. Compensation of student test subjects for participation through test subject hours *or* test subject fees (10€/h).
9. Follow-up measurement takes place after 4 weeks instead of after 3 months, as the study must be completed within a teaching term.

Literature:

Barbor, T.F., Higgings-Biddle, J.C., Saunders, J.B. & Monteiro, M.G. (2001). AUDIT – The alcohol use disorder identification test. guidelines for use in primary Care. 2nd edition. Geneva: World Health Organization.

Gollwitzer, P.M. (2012). Mindset theory of action phases. In P. Van Lange, A.W. Kruglanski & E.T. Higgins (Eds.), *Handbook of Theories of Social Psychology* (pp. 526-545). London: Sage Publications.

Humeniuk, R., Henry-Edwards, S., Ali, R., Poznyak, V., & Monteiro, M. G. (2010). The ASSIST-linked brief intervention for hazardous and harmful substance use: a manual for use in primary care. Geneva: World Health Organization.

Josef, A.K., Richter, D., Samanez-Larkin, G.R., Wagner, G.G., Hertwig, R., Mata, R. (2016). Stability and change in risk-taking across the adult life span. Journal of Personality and Social Psychology, Vol. 111(3), 430-450.

Sobell, L. C., & Sobell, M. B. (1992). Timeline follow-back: A technique for assessing selfreported alcohol consumption. In R.Z. Litten & J. Allen (Eds.). *Measuring alcohol consumption: Psychosocial and Biological Methods* (pp. 41-72). New Jersey: Humana Press.

Annex 3.1

Declaration of consent that is displayed when potential participants register for this study at SONA

Important Human Subject and Privacy Policy Notice
Both the test subject and the investigator must agree to the following guidelines for conducting experiments with humans and for data protection before they are allowed to make use of the Study Pool of the University of Konstanz. In addition, both the investigator and the test subject commit themselves to treat each other respectfully. If you have any questions about policies, rights and responsibilities, please contact: sona@uni-konstanz.de.

Characteristics of psychological experiments

Psychological research depends on the participation of humans as test subjects. Psychologists are aware of the special nature of the relationship between investigators and test subjects and the resulting responsibility. They shall ensure that research respects the dignity and integrity of participants. They shall take all appropriate measures to ensure the safety and well-being of persons participating in research and shall seek to exclude any risks.

Data protection

The data recorded in this system are used exclusively for the organization of scientific experiments. The data will not be passed on to third parties and will only be used for the following purposes:

- Invitation of participants to experiments
- Carrying out scientifically motivated selection of persons to participate in specific experiments
- Checking the participation or non-participation of registered individuals during experiments

Furthermore, there is no link between the data collected in the respective experiments and the data in the study pool (with the exception of the data from the pre-survey, which were made available to the experimental management).
Information on the participation of test subjects in studies is only accessible to the respective investigator and the administrators of SONA. However, if there is any suspicion of abuse of privacy, please contact sona@uni-konstanz.de.

Rules of Conduct (Rules of Conduct)

Investigators as well as test subjects commit themselves to a respectful mutual interaction. Statements that violate the personal rights of test subjects or investigators are to be omitted. These include in particular sexual molestation and sexist or racist insults. Violation of these rules of mutual conduct will result in exclusion from the database.
If you believe that someone has violated these basic rules, please email us at sona@uni-konstanz.de.

Rights of use

- Participation in experiments is entirely voluntary and can be terminated at any time without giving reasons and without any disadvantages. In addition, collected data can be deleted at the request of the participant.
- As long as the right to terminate participation is not exercised, the test subjects have to follow the instructions of the investigator. Failure to follow the instructions may result in exclusion from experiment and payment.
- If one decides to answer the questions about age and gender in the preliminary survey, then the information given has also to be true. Otherwise, deliberate deception may lead to exclusion from the database.

Miscellaneous

The person who registers for an experiment has also to be the person who appears for the experiment. Registrations for other persons are strictly prohibited. In some cases, test participants are requested by the experimenter to provide a valid identification.

Annex 3.2

Declaration of consent to the study at Unipark:

Information and declaration of consent
Study: Alcohol Risk Perception ***Studienleitung***: Dr. Michael Odenwald (University of Konstanz)
Aim and purpose of the study: In this study, we examine individuals with regard to their willingness to change their alcohol consumption. Our aim is to gain a better understanding of alcohol-related risk perception.
Procedure: You will receive questions about yourself, your alcohol and other substance consumption as well as your perception of your alcohol consumption and the associated risks. You will need about 15-20 minutes today. Some of the participants will be invited to further appointments at a later date.
Risks: The investigation does not include any risks or dangers.
Service/costs: You will not be charged any costs for the examination. You will receive either half a *test subject hour* a or 5€ for the participation. At the end of the study, you can choose between the two forms of remuneration.
Data protection: The information you disclose is subject to confidentiality. All information will be kept strictly confidential. They are stored in locked cabinets and are not passed on to third parties. Only authorized employees have access to the data collected. The information is stored anonymously on secure computers. The analysis of the data does not allow any conclusions to be drawn about your person.
Other: Participation in the study is voluntary. You can decide not to participate at any time without being disadvantaged. By your consent you do not enter into any obligations. You may cancel your participation at any time and without giving any reason, without any disadvantage.
Do you have any questions? If so, please contact michael.odenwald@uni-konstanz.de.

By clicking on "I would like to participate" you agree to the following: I volunteer to participate in the investigation. I know I can withdraw my participation in the investigation at any time. I have read the preceding declaration. I know that it is not allowed to pass on data about my person to third parties, that they are stored and processed anonymously and that from possible scientific publications no conclusions about my person can be drawn. I know that the investigation does not involve any risks.
☐ I would like to participate!
☐ No, I do not want to participate.

Annex 4

Additional items from the Socioeconomic Panel

Human behavior may vary in different domains. How would you rate your readiness to risky behavior in the following domains?
How risk-prone are you….

	Not at all risk-prone									Very risk-prone
… trusting other people?	○	○	○	○	○	○	○	○	○	○
… driving a car?	○	○	○	○	○	○	○	○	○	○
… in leizure time and sports?	○	○	○	○	○	○	○	○	○	○
… related to financial in-vestments?	○	○	○	○	○	○	○	○	○	○
… related to your own health?	○	○	○	○	○	○	○	○	○	○
… related to your career?	○	○	○	○	○	○	○	○	○	○

Information from the website of the Max Planck Institute for Human Development:
<font color="#ffff00">-=https://www.mpib-berlin.mpg.de/de/presse/2016/01/risikobereitschaft-veraendert-sich-in-jungen-jahren-und-im-hoeheren-alter-am-staerksten=- proudly presents

The Socio-Economic Panel (SOEP) is a representative multi-cohort survey conducted since 1984. For the studies conducted under the auspices of the Leibniz Association at DIW Berlin, every year about 30,000 people in almost 15,000 households in Germany are interviewed by TNS Infratest Social Research (Munich). For example, they provide information on personal attitudes and political views, income, employment, education or health. As the same people are interviewed every year, long-term psychological, economic, social and societal developments can be identified.

Universität
Konstanz

Universität Konstanz · Box 214 · 78457 Konstanz

Sabine Schieß
Institutional review board
(Ethics Commission)
Universitätsstraße 10
D-78464 Konstanz
+49 7531 88-5264
Fax +49 7531 88-3727

sabine.schiess@uni-konstanz.de
www.uni-konstanz.de

Herr
Dr. Michael Odenwald
im Hause

30.11.2017

IRB statement 21/2016
and amendment (23.10.2017)

Seite: 1/1

To whom it may concern,

in my function as Secretary of the Institutional Review Board (Ethics Committee) of the University of Konstanz I would like to deliver the following statement regarding the research project **"Research Group RISKDYNAMICS, Sub-Project 8, Work Package 1, Study 2 (Student Sub-study): Addiction Risk: The Influence of Mindset Induction on the Effect of a Brief Intervention to Reduce Alcohol Use"** which was reviewed at the University of Konstanz as the relevant and most proximate academic institution with an standing institutional review board:
The IRB of the University of Konstanz has evaluated the above proposal, and we have come to the conclusion that this project, and the procedures for data handling and storage are in full line with the ethics regulations of the University of Konstanz.
This judgement was made on following basis:

- the text of the proposal,
- the specific regulations of the University for ethical experimentation and data storage,
- Declaration of Helsinki in its current version as developed by the World Medical Association,
- Relevant national and international law and regulations

The University has an administrative procedure in place that will ensure that ethics guidelines as laid down in German national law and international conventions are fully respected. The IRB will ensure and, if necessary, enforce the proper adherence to ethics guidelines during and after the project.
This IRB approval is given for five years for the specific evaluated project. This also includes additions and prolongations of the project of a technical nature that do not affect the overall judgement of ethical issues and the state of safety of the study subjects.
Sincerely,
Sabine Schieß
