# Supplementary material for: The effects of pre-intervention mindset induction on a brief intervention to increase risk perception and reduce alcohol use among university students: A pilot randomized controlled trial
Source: PLoS One. 2020 Sep 17;15(9):e0238833. doi: 10.1371/journal.pone.0238833 (PMC7498304; doi:10.1371/journal.pone.0238833)
Supplement: Attachment — Submitted filename: Response to reviewers.docx [file pone.0238833.s004.docx]

**Response to reviewers**

**Vanessa Carels (Editor)**

Comment E.1: *Please ensure that your manuscript meets PLOS ONE's style requirements, including those for file naming.*

Response: We took great care to meet *PLOS ONE*’s style requirements.

Comment E.2: *Please provide additional details regarding participant consent.*

*In the ethics statement in the Methods and online submission information, please ensure that you have specified (a) whether consent was informed and (b) what type you obtained (for instance, written or verbal, and if verbal, how it was documented and witnessed).*

*If your study included minors, state whether you obtained consent from parents or guardians. If the need for consent was waived by the ethics committee, please include this information.*

Thank you. The paragraph on ethical approval was extended and reads now as follows (Lines 173 – 193):

“The study protocol was approved by the Institutional Review Board of the University of Konstanz, Germany; the trial registry number is NCT03338491 ([www.ClinicalTrials.gov](http://www.ClinicalTrials.gov)). According to the IRB approval participants of the screening gave informed consent by clicking the respective button in the experiment management system. All participants of the intervention study gave written informed consent. All intervention study participants were fully informed about the study after completing the follow-up assessment.”

Comment E.3: *We note that you have stated that you will provide repository information for your data at acceptance. Should your manuscript be accepted for publication, we will hold it until you provide the relevant accession numbers or DOIs necessary to access your data. If you wish to make changes to your Data Availability statement, please describe these changes in your cover letter and we will update your Data Availability statement to reflect the information you provide.*

Thank you for this information. We are aware of this procedure.

Comment E.4: *Please include captions for your Supporting Information files at the end of your manuscript, and update any in-text citations to match accordingly. Please see our Supporting Information guidelines for more information: http://journals.plos.org/plosone/s/supporting-information*

Thank you. We now included one table into the Supporting Information and labeled it “Table S1”.

**Reviewer #1**

Comment 1.1: *Thank you for your paper, it is an interesting read, and important for the field. It is a well written paper, and I have the following minor comments to make:*

Thank you! We took great care to incorporate your thoughts and comments in revising the manuscript, and we hope that you see our revisions as an improvement to the manuscript.

Comment 1.2: *Abstract:*

*Line 23, "showed" rather than "did show"*

*Introduction:*

*Line 45, "perception is a key factor" rather than "as a key factor"*

*Line 180: should it read "I intend to do!"?*

We combined these comments as they pertain to grammar issues or typos. We changed the manuscript accordingly, thank you!

Comment 1.3: *SBIs vary markedly in their components and length. Can citations be included with specific details as to which have been found most effective, and in particular for students as your group of interest.*

We added some information and a citation of a review that aimed at identifying the mechanisms of effective college drinking interventions in lines 98 - 100: “A meta-analysis was able to show that most interventions with significant effects on college drinking were delivered face-to-face by skilled professionals while data on the length of the intervention were inconclusive (Carey et al., 2016).”

Comment 1.4: *Lines 116-117: At what time point was recruitment stopped? By how much did the alcohol use increase, hence halting recruitment?*

Recruitment of student participants is only possible during the teaching terms (semesters) as many students do not spend their breaks in Konstanz and thus would not be available for study participation, let alone for any study involving follow-ups. We had planned to conduct the study over a whole year (i.e., spanning two semesters; Nov. 2018 to Oct. 2018) in order to reach the intended number of participants (120). After the end of the first semester in February 2018, students who wrote their master theses in this project analyzed the data collected up to this point and found the results that we report in the submitted manuscript. We now mention the originally planned recruitment period and the actual date of stopping recruitment (lines 163 – 172). The average increase of about 7 standard drinks (in relation to the 43 standard drinks reported at t1) in the deliberative mindset condition is the alcohol use increase that halted recruitment.

Comments 1.5: *Line 204: who trained the students? Trained in what respect?*

Thank you, we now provide more information regarding your question. The manuscript reads in lines 289 – 292: “The total duration of the ASSIST-linked Brief Intervention was about 30 min and was conducted by postgraduate psychology students who were intensively trained by licensed psychotherapists using a combination of theoretical and practical methods, including role plays and on site supervision.”

**Reviewer #2**

Comment 2.1: *This is an interesting and elegantly designed study yielding valuable results concerning the unexpected effects of induction of deliberative versus implemental mindsets among university students with risky drinking, who all received the ASSIST brief intervention following the induction. The manuscript is generally clear but some clarifications are needed and, mainly, a thorough English-language review. See general and specific comments below.*

Thank you for your generally positive evaluation of our manuscript. We took great care to adequately respond to your comments. We hope that our revised manuscript will meet your expectations.

Comments 2.2: *General Comments:*

Comment 2.2.1: *The abstract needs to be clarified somewhat, see below. ´*

We will address this in Comment 2.3.2.

Comment 2.2.2: *The definition of risky drinking differs between men and women, as indicated in the first paragraph of the introduction. The authors do not report any gender-related analyses, however. Please complement the results with information on any gender differences, as well as information on the percentage of participants (by gender) over the risky drinking level at t2; the reviewer’s understanding is that 100% were risky drinkers at t1.*

Let us first address the issue of the classification of risky drinking. We used the AUDIT to screen for risky drinking at t0. Based on their AUDIT score of eight or above, the participants invited to take part in t1 and t2 were considered risky drinkers. Using a general cutoff score of eight (as usually used in the literature), we did not discriminate between genders. All participants of the trial reported here were risky drinkers based on this criterion. After the screening, the AUDIT was not employed any further. The main outcome of the SBI was determined using the TLFB, i.e., a pre-post comparison of consumed alcohol units. Thus, the refinement in assessing alcohol consumption (i.e., a more fine-grained method like the TLFB vs. a broader, more time-efficient approach in the prescreening like the AUDIT) makes a direct comparison of percentages rather uninformative. So again, all participants of t1 and t2 were risky drinkers according to their AUDIT scores (which assess drinking behavior over the past year and thus were unlikely to change over the course of the experiment). When using gender-specific limits for the four weeks that we observed at t1 (i.e., 50 standard units for men vs. 30 standard units for women), 53% of all participants, that is, 56% of women and 44% of men could be classified as risky drinkers. Further refinements of this classification could include actual drinking patterns (e.g., more than 5 consecutive drinking days) but because standard units consumed is the main outcome variable of the present article, this should be the scope of another research article.
In regard to gender differences and effects, we want to exert caution. Because only 18 of the 66 participants of t1 were men, all the following should be taken with a grain of salt.

We included gender as factor in the rmANOVAs of all outcome variables. There was no significant threefold interaction term (time*mindset*gender) and no significant twofold interaction term involving gender*mindset. Several outcome variables were significantly influenced by an interaction term (time*gender), i.e., FAR-Perceived Personal Vulnerability, SOCRATES-Recognition and SOCRATES-Taking Steps. Gender showed a main effect on URICA-Precontemplation. In the rmANOVA with TLFB as dependent variable gender did not have any significant effect.

In sum, our data suggest that gender was not a moderator of the observed mindset effects, it did not change the overall picture of the results, and there was only a low number of men in our sample. We thus refrained from placing a strong emphasis on gender differences and effects in the manuscript. However, we added information on any gender effects in the respective paragraphs (e.g., lines 352 – 353). If you think it would be necessary, we would prepare more details on the effects of gender in a supplementary material.

Comment 2.2.3: *The conceptual model linking mindset, risk perception, readiness to change, the intervention consisting of PF and decisional balance, and the alcohol use outcome, would be clearer if a figure were provided, either in the introduction or in the discussion. Indeed, it is not entirely clear why alcohol use was not designated as the sole primary outcome, notwithstanding the authors’ review of earlier research supporting risk perception and readiness to change as moderators of alcohol use outcomes following intervention.*

This is a very good suggestion, however, while we think that although theoretical models exist that justify the inclusion of the mentioned variables, an overarching model that includes all of them needs to be developed. We would like to be cautious about preliminarily reporting such a model before empirical evidence has been accumulated.

Comment 2.2.4: *In the method section. The mindset induction is clearly described. However, it is stated that “a cover story was used…”. Please explain the rationale for the cover story and describe the instructions given.*

The cover story was necessary to conceal the relation of the mindset induction with the SBI in order to prevent participants from forming own theories about the type of relation between the both intervention components. As it is common that students take part in multiple studies, we created a cover story that mindset induction and alcohol intervention belong to unrelated different studies. After the follow-up assessment, participants were fully informed about the research design and research questions.

Lines 152 – 156: “A cover story was used that suggested that the mindset induction was unrelated to the rest of the study. More specifically, participants were told that the experimenter needed to prepare for the upcoming part of the experiment and that the participant could use this time by completing a questionnaire. This questionnaire (i.e., the mindset manipulation) had its own informed consent and stated that it was designed by another group of researchers (i.e., the social psychology and motivation group).”

Comment 2.2.5: *The measures are adequately described, but it is not clear whether the German versions have been validated or simply translated. Please clarify. Also, it is not clear how resistance was measured by the student counselors, and whether any inter-rater reliability procedure was used (if not, include in limitations in the discussion).*

Thank you. We used instruments whose German versions had been validated. We now consequently cite the respective German validation studies.

Lines 251 and 252 now clarify why there is no inter-rater reliability for this measure. Lines 463 – 465 address this issue in the discussion.

Comment 2.2.6: *The results are also adequately described, but they are not presented in the order of the primary and secondary outcomes specified in the manuscript as well as in the trial registration description. The risk perception and alcohol use outcomes should be presented first, and the readiness to change outcomes should be presented afterwards, as the secondary outcomes.*

Thank you, we changed the order as you suggested.

Comment 2.2.7: *Table A1, showing the t2 study outcomes, should be included in the manuscript as Table 2, not in the Appendix. (Table A2 can remain in the Appendix).*

Thank you, we included Table A1 as Table 2 in the manuscript.

Comment 2.2.8: *The discussion of the deliberative and implemental mindsets as related to the unexpected outcomes on alcohol use is interesting and well-informed. However, the discussion of the implemental mindset results could be revised to include the motivational interviewing (MI) approach described in “Ten strategies for evoking change talk”, namely asking the client to describe previous successful experiences of making a decision to change.*

Thank you for reminding us of this list. In our eyes, the inclusion of the “Ten strategies for evoking change talk” is not necessary for our arguments. But we added a general suggestion regarding training referring to this and other training materials.

Lines 547 – 549: “A standardized training would help to implement the different MI skill levels of therapists, e.g. a training for using the different methods to evoke change talk or to avoid sustain talk.”

Comment 2.2.9: *Regarding the discussion of future studies, factorial design (LM Collins, Optimization of Behavioral, Biobehavioral, and Biomedical Interventions The Multiphase Optimization Strategy (MOST), 1st ed. 2018. ed. Cham: Springer International Publishing : Imprint: Springer, 2018) could be an ideal way of evaluating the questions posed, in a design evaluating mindset (deliberative vs implemental vs none), MI skills (high vs low), Personal feedback (yes/no) and decisional balance (yes/no). Factorial designs, although complex, identify optimal components to include in interventions (e.g., Crane D, Garnett C, Michie S, West R, Brown J. A smartphone app to reduce excessive alcohol consumption: Identifying the effectiveness of intervention components in a factorial randomised control trial. Scientific reports. 2018;8(1):4384.). A larger sample would be needed, but not as large as a traditional randomized controlled trial.*

Thank you for this helpful suggestion that is especially valuable for our next research project. We agree that the design you propose would be perfect for answering a host of research questions. As a 3x2x2x2 (i.e., 24 conditions) design with the potential of four-way interactions, we find it too complicated to be only mentioned in passing. As we already describe the independent variables and hypotheses of an exemplary future study, we think that adding the specific design would be a too detailed piece of information in this manuscript.

Comments 2.3 – *Specific Comments:*

Comment 2.3.1: *The title is a bit unclear. Perhaps:*

*Brief intervention to increase risk perception and reduce alcohol use among university students: A pilot randomized controlled trial evaluating the effects of pre-intervention mindset induction*

We changed the title in response to your comment. We included the pre-intervention qualifier to clarify the role of the mindset induction.

Comment 2.3.2: *The abstract is also imprecise:*

*• Line 10. Effects, not consequences*

*• Lines 10-11 very complex sentence, revise. “the openness and thereby the effectiveness…” gives unnecessary information in the abstract and is confusing.*

*• Line 14. Better outcomes – better than what?*

*• Line 15. Sample of 257 students, not 256.*

*• Lines 15-18, revise: “A sample of 257 students provided baseline measures on risk perception, alcohol use and readiness to change, after which 63 students with risky alcohol use were randomly allocated to one of two mindset induction conditions – deliberative or implemental. Then, they received an in-person ASSIST-linked brief inyervention and completed self-report questionnaires on changes in risk perception, alcohol use, and readiness to change at post-intervention and four-week follow-up.”*

*• Lines 23-24. English: should be “neither….showed any changes in risk perception or in readiness to change…”*

*• Line 27. “the processes involved” is too vague; specify.*

Thank you for your many and helpful comments to the abstract. We tried to incorporate all your remarks and hope that the new abstracts fits your expectations.

Comment 2.3.3: *Introduction*

*• Lines 67-68, delete “Whereas”; enough to say “Once the decision is made…”.*

Thank you! We followed your suggestion and deleted the word.

Comment 2.3.4: *Method*

*• Line 92, should be “in which one of two mindets…” to be clear.*

*• Line 98, delete “just”, add “on the cover of the envelope”.*

*• Lines 102-104, explain “cover story”, see above.*

*• Lines 110-111, can be part of the paragraph above.*

*• Line 113. Should read: “sample size…would be required…”*

*• Participants – adjust English.*

*• Line 128. “second appointment” – meaning what? T2?*

Thank you for highlighting these parts where further clarifications were needed. We adjusted the manuscript accordingly.

Comment 2.3.5: *Results*

*• Lines 253-254, a bit too much information? Personal vulnerability earlier described as “own vulnerability”, keep it consistent. Personal or self better than “own”.*

*• Lines 261-262. Do not include non-significant results or trends.*

We now use “(perceived) personal vulnerability” throughout the manuscript and further shortened the sentence. Regarding the second part of your comment, we now highlight that the comparison between t1 and t2 in the implemental mindset condition constitutes only a trend. Because it is essential to understand the interaction between mindset condition and time, we think that we still had to include it at this point.

Comment 2.3.6: *Discussion*

*• Lines 323-324. Should read “These authors evaluated a brief MI intervention aimed to reduces alcohol use among young heavy drinkers and found that inexperienced therapists…”*

*• Line 373. Emerge, not evince.*

*• Line 378. Not necessarily so that risk perception would decrease after implementing alcohol use reduction. Perhaps personal vulnerability would decrease, but the risk perception should actually have increased, not decreased. Please clarify.*

We addressed the issues you raised in this comment and clarified our sentence in the discussion. Lines 561 – 562 now read: “…in the case of implemented alcohol use reduction, alcohol risk perception (especially the domain perceived personal vulnerability) should eventually decrease”

Comment 2.3.7: *Table 1*

*• Test statistic not necessary to include.*

We dropped the test statistic. Thank you for this remark!

Comment 2.3.7: *Although the writing is generally clear, the journal does not provide copy-editing and the English should be reviewed by a native speaker with scientific editing skills.*

Thank you for all of your comments and suggestions. We are sorry for any mistakes that were made.
