# Supplementary material for: The effects of pre-intervention mindset induction on a brief intervention to increase risk perception and reduce alcohol use among university students: A pilot randomized controlled trial
Source: PLoS One. 2020 Sep 17;15(9):e0238833. doi: 10.1371/journal.pone.0238833 (PMC7498304; doi:10.1371/journal.pone.0238833)
Supplement: Attachment — Submitted filename: renamed_5a96c.docx [file pone.0238833.s005.docx]

**COMMENTS – Manuscript PONE-D-19-18841R1**

Title: “The effects of pre-intervention mindset induction on a brief intervention to increase risk perception and reduce alcohol use among university students: A pilot randomized controlled trial”

**Important note: This review pertains only to ‘statistical aspects’ of the study and so** **‘****clinical aspects’ [like medical importance, relevance of the study,** **‘clinical significance and implication(s)’ of the whole study, etc.] are to be evaluated [should be assessed] separately/independently.**

It is definitely a well written article; however, there are few questions and I have the following minor comments to make:

Your ABSTRACT is well drafted but assay type. Please note that it is preferable [refer to item 1b of CONSORT checklist 2010: Structured summary of trial design, methods, results, and conclusions] to divide the ABSTRACT with small sections like ‘Objective(s)’, ‘Methods’, ‘Results’, ‘Conclusions’, etc. which is a accepted practice of most good/standard journals [including PLOS]. It will definitely be more informative then, I guess.

Please check {for a possible} typing error in line 120 [The trial started in the winter term 2017/18, recruitment was originally planned for two subsequent semesters between November 2017 and October 2012 and t2 assessments were planned to be terminated before the end of the teaching term.].

To provide a description of baseline characteristics is entirely reasonable (since it is clearly important in assessing to whom the results of the trial can be applied), however, it does not require the division of baseline characteristics by treatment groups {lines 223-4: To test for baseline differences between mindset groups, χ²-tests were performed for categorical variables and univariate ANOVAs for continuous variables}. Statistical comparison of baseline characteristics [last ‘P-value’ column in Table 1] is not desirable at all [because even if P-value turns out to be significant (while comparing baseline characteristics despite random allocation), it is, by definition, a false positive] as you are supposed to be testing ‘randomization’ then, which in any single trial may not balance all baseline characteristics because ‘randomization’ is a sort of ‘insurance’ and not a guarantee scheme.

References:

1. Stuart J. Pocock, et al., ‘Subgroup analysis, covariate adjustment and baseline comparisons in clinical trial reporting: current practice and problems’, Statistics in medicine, 2002; 21:2917–2930 [Particularly page 2927]
2. Harrington D, et al., ‘New guidelines for statistical reporting in the journal’, N Engl J Med 2019;381:285-6

[Important message from these articles: Never do any comparison with respect to ‘baseline’ characteristics].

Because for missing data due to dropout (two participants), the last observation carried forward method was chosen [lines 218-9], please note (must be known to these learned authors, still may please be noted) that according to available literature [example, “Inference and Missing Data,” Biometrika, 1976, vol:63, 581–592 and “Multiple Imputation After 18+ Years,” Journal of the American Statistical Association, 1996, vol:91, 473–489] ‘Multiple Imputation’ technique [procedure replaces each missing value with a set of plausible values that represent the uncertainty about the right value to impute. These multiply imputed data sets are then analysed by using standard procedures for complete data and combining the results from these analyses. It is claimed that ‘No matter which complete-data analysis is used, the process of combining results from different imputed data sets is essentially the same’. This results in valid statistical inferences that properly reflect the uncertainty due to missing values], is preferred [considering MCAR (Missing Completely At Random) expected nature of data] than {despite being time-consuming and involving much more computations} compare to all out of other important imputation techniques frequently used [like Group Means, Hot-deck Imputation, Baseline Observation Carried Forward (BOCF), Worst Observation Carried Forward (WOCF), Predicted Mean, and even Last Observation Carried Forward (LOCF)].

When you say [line 23] that “In contrast to our hypotheses, …….” and later in lines 82-91 (not pasted) & in lines 307-9 [We had expected that the induction of a deliberative versus an implemental mindset would enhance the acceptance of the alcohol feedback and, thus, increase participants’ risk perceptions and readiness to change, leading to reductions in alcohol consumption], remember the principle of ‘*equipoise*’ [which means that there is genuine uncertainty in the expert medical community over whether a treatment will be beneficial. An ethical dilemma arises in a clinical trial when the investigator(s) begin to believe that the treatment or intervention administered in one arm of the trial is significantly outperforming the other arms. A trial should begin with a null hypothesis, and there should exists no decisive evidence that the intervention or drug being tested will be superior to existing treatments or effective at all. Infact equipoise is a sort of assurance that there is no prejudice and it is to avoid Asher’s paradox.].

While saying [in lines 26-27] “neither deliberative nor implemental mindset participants showed any changes in risk perceptions or in their readiness to change alcohol consumption” remember that “Absence of evidence is not evidence of absence” [Altman DG, Bland JM. BMJ volume 311, 1995, p 485 (Reprinted : Australian Veterinary Journal 1996;74, 311)]. Absence of evidence implies that null hypothesis of no difference is not rejected i.e. result is not significant but that does not amount to evidence of absence i.e. evidence of ‘no difference’.

Justification {or account given otherwise} on sample size given in lines 122-27 was not necessary because this being a pilot study sample size is not a vital issue anyway. As said in lines 169-70 [For SOCRATES and the URICA Precontemplation Scale, we modified the original Likert answer scales into a visual analogue scales to prevent memory biases] can you give more explanation on ‘how modification of the Likert answer scales into a visual analogue scales’ prevent memory biases? (and what these memory biases are)?

Note that non-parametric parallel of one-way ANOVA is ‘Kruskal-Wallis’ test and not Mann-Whitney test [as said in lines 224-5: If Levene tests revealed heterogeneity of variances, Mann-Whitney tests were used instead.]. Mann-Whitney test is for comparing two groups and parallel to ‘t’ test for two independent groups. In this trial there are two independent groups and application of Mann-Whitney test [when Levene test revealed heterogeneity of variances] is correct. But when there are two independent groups to be compared ‘why apply ANOVA’ which is for comparison of more than two groups? {it is true that ‘if there are only two independent groups ‘ANOVA’s “F” is square of ‘t’ but logic and so algorithm are different}. Whenever there are two independent groups we never apply ANOVA, though ‘F’ and ‘t’ are mathematically similar. Please explain if the reason is what said in lines 231-2 [“We chose ANOVAs as many findings speak for the robustness of the analysis of variance concerning violated assumptions”], {what is that?}.

I wish that a lager study over coming limitations of the present study [as pointed out in lines 400-410] should be conducted by the same team of scientists.
