# Supplementary material for: The effects of pre-intervention mindset induction on a brief intervention to increase risk perception and reduce alcohol use among university students: A pilot randomized controlled trial
Source: PLoS One. 2020 Sep 17;15(9):e0238833. doi: 10.1371/journal.pone.0238833 (PMC7498304; doi:10.1371/journal.pone.0238833)
Supplement: Attachment — Submitted filename: 200818 - Response to Reviewers_final.docx [file pone.0238833.s006.docx]

**Response to reviewers**

**Dr Gillian W. Shorter (Editor)**

Comment E.1: *Could you thoroughly check that you have addressed the full CONSORT statement, checking the elaboration statement that the required detail is present. I will check this carefully on resubmission. Thank you.*

Response: We took great care to address the CONSORT statement. We very much hope that our revised manuscript will meet your expectations. Thank you so far for your support and helpful suggestions.

In the process of the revision we noticed an incongruity in the tables. We reported the means and standard deviation of the sum scores of some variables (DOSPERT, FAR) in the tables, but we computed the reported analysis with the average scores of the variables. We changed the values in the tables, so they are now consistent with the analysis reported in the text. We are very sorry for this mismatch.

**Anne H Berman (Reviewer #2)**

Comment 2.1: *I am satisfied with the authors' responses to my comments as well as the changes made in the manuscript.*

Response: Thank you! We are glad that you are satisfied with our revisions and we are very thankful for the constructive suggestions you made and for your positive evaluation.

Comment 2.2: *The language is improved, but I did notice an error on p. 13, line 463 where the word "of" is missing ("Please note, however, that because how we designed..."; should be "Please note, however, that because of how we designed..."). There may be additional similar minor errors so a final proofreading is probably a good idea. Good luck with your future research!*

Response: We corrected it accordingly and we will conduct a final proofreading, thank you for your advice!

**Dr. Sanjeev Sarmukaddam (Reviewer #3)**

Comment 3.1: *Important note: This review pertains only to ‘statistical aspects’ of the study and so ‘clinical aspects’ [like medical importance, relevance of the study, ‘clinical significance and implication(s)’ of the whole study, etc.] are to be evaluated [should be assessed] separately/independently.*

*It is definitely a well written article; however, there are few questions and I have the following minor comments to make:*

Response: Thank you for your evaluation. We took great care to adequately respond to your questions and comments and we hope that our revised manuscript will meet your expectations.

Comment 3.2: *Your ABSTRACT is well drafted but essay type. Please note that it is preferable [refer to item 1b of CONSORT checklist 2010: Structured summary of trial design, methods, results, and conclusions] to divide the ABSTRACT with small sections like ‘Objective(s)’, ‘Methods’, ‘Results’, ‘Conclusions’, etc. which is a accepted practice of most good/standard journals [including PLOS]. It will definitely be more informative then.*

Response: Thank you for this advice. We divided the abstract into small sections as you suggested.

Comment 3.3: *Please check {for a possible} typing error in line 120 [The trial started in the winter term 2017/18, recruitment was originally planned for two subsequent semesters between November 2017 and October 2012 and t2 assessments were planned to be terminated before the end of the teaching term.].*

Response: Indeed, it should mean “2018“ instead of “2012”. We corrected the typing error accordingly, thank you!

Comment 3.4: *To provide a description of baseline characteristics is entirely reasonable (since it is clearly important in assessing to whom the results of the trial can be applied), however, it does not require the division of baseline characteristics by treatment groups {lines 223-4: To test for baseline differences between mindset groups, χ²-tests were performed for categorical variables and univariate ANOVAs for continuous variables}. Statistical comparison of baseline characteristics [last ‘P-value’ column in Table 1] is not desirable at all [because even if P-value turns out to be significant (while comparing baseline characteristics despite random allocation), it is, by definition, a false positive] as you are supposed to be testing ‘randomization’ then, which in any single trial may not balance all baseline characteristics because ‘randomization’ is a sort of ‘insurance’ and not a guarantee scheme.*

*References:*

*1. Stuart J. Pocock, et al., ‘Subgroup analysis, covariate adjustment and baseline comparisons in clinical trial reporting: current practice and problems’, Statistics in medicine, 2002; 21:2917–2930 [Particularly page 2927]*

*2. Harrington D, et al., ‘New guidelines for statistical reporting in the journal’, N Engl J Med 2019;381:285-6*

*[Important message from these articles: Never do any comparison with respect to ‘baseline’ characteristics].*

Response: You made a very good point that is especially valuable for our subsequent research project. In clinical research, especially when conducting RCTs, it is a common practice to compare baseline characteristics between treatment groups to be able to interpret results correctly. In this pilot study that reports unexpected findings, we want to be especially clear about the possible case that potentially undetected baseline differences might have overlaid the mindset effects – a respective reviewer comment would have been inevitable if we wouldn’t have reported on baseline characteristics on a subgroup level.

Comment 3.5: *Because for missing data due to dropout (two participants), the last observation carried forward method was chosen [lines 218-9], please note (must be known to these learned authors, still may please be noted) that according to available literature [example, “Inference and Missing Data,” Biometrika, 1976, vol:63, 581–592 and “Multiple Imputation After 18+ Years,” Journal of the American Statistical Association, 1996, vol:91, 473–489] ‘Multiple Imputation’ technique [procedure replaces each missing value with a set of plausible values that represent the uncertainty about the right value to impute. These multiply imputed data sets are then analysed by using standard procedures for complete data and combining the results from these analyses. It is claimed that ‘No matter which complete-data analysis is used, the process of combining results from different imputed data sets is essentially the same’. This results in valid statistical inferences that properly reflect the uncertainty due to missing values], is preferred [considering MCAR (Missing Completely At Random) expected nature of data] than {despite being time-consuming and involving much more computations} compare to all out of other important imputation techniques frequently used [like Group Means, Hot-deck Imputation, Baseline Observation Carried Forward (BOCF), Worst Observation Carried Forward (WOCF), Predicted Mean, and even Last Observation Carried Forward (LOCF)].*

Response: You are completely right, thank you for your advice. We followed your suggestion and computed the whole analysis using multiple imputation instead of the Last Observation Carried Forward Method. The numeric results changed very little, the outcome of statistical tests stayed the same with no further implications for the interpretation. Line: 223-224 (lines refer to the manuscript document without highlighted changes): “For replacement of missing data due to dropout (two participants), the multiple imputation technique was used (Little’s MCAR test, χ² (22) = 28.24, *p* = .168).” And results are reported below (ff. line 260). We hope to meet your expectation on that point.

Comment 3.6: *When you say [line 23] that “In contrast to our hypotheses, …….” and later in lines 82-91 (not pasted) & in lines 307-9 [We had expected that the induction of a deliberative versus an implemental mindset would enhance the acceptance of the alcohol feedback and, thus, increase participants’ risk perceptions and readiness to change, leading to reductions in alcohol consumption], remember the principle of ‘equipoise’ [which means that there is genuine uncertainty in the expert medical community over whether a treatment will be beneficial. An ethical dilemma arises in a clinical trial when the investigator(s) begin to believe that the treatment or intervention administered in one arm of the trial is significantly outperforming the other arms. A trial should begin with a null hypothesis, and there should exists no decisive evidence that the intervention or drug being tested will be superior to existing treatments or effective at all. Infact equipoise is a sort of assurance that there is no prejudice and it is to avoid Asher’s paradox.].*

*While saying [in lines 26-27] “neither deliberative nor implemental mindset participants showed any changes in risk perceptions or in their readiness to change alcohol consumption” remember that “Absence of evidence is not evidence of absence” [Altman DG, Bland JM. BMJ volume 311, 1995, p 485 (Reprinted : Australian Veterinary Journal 1996;74, 311)]. Absence of evidence implies that null hypothesis of no difference is not rejected i.e. result is not significant but that does not amount to evidence of absence i.e. evidence of ‘no difference’.*

Response: Thank you. You describe accurately the theoretical approach that should be the guiding philosophy of experimental designs.

Re principle of ‘equipoise’: In writing clinical manuscripts, we are of course familiar with the convention that researchers have to be impartial; but if the existing literature favors a directed alternative hypothesis it is preferred over a undirected alternative hypothesis. In this case, we preferred a directed alternative hypothesis and stated this clearly in the trial registration. However, our experiment did not support our pre-assumptions. We hope it is made clear in the discussion that we learned a lot by this experiment, much of what you pointed out.

Re interpretation of findings: We are aware that the absence of evidence does not mean that there is no mindset effect at all. But having regard to your note, we added the following sentence for clarification in the discussion [lines 409 - 413]: “But although no support was found for our hypotheses that the specific mindset during an intervention has an influence on the change of the variables risk perception and readiness to change alcohol consumption, it does not necessarily imply that there are no mindset and intervention effects on these variables. It would be premature however to conclude that these variables were unresponsive as we did not study their trajectories.”

Comment 3.7: *Justification {or account given otherwise} on sample size given in lines 122-27 was not necessary because this being a pilot study sample size is not a vital issue anyway.*

Response: Thank you for your advice. We agree that in a pilot study the calculation of sample size is not necessary. But we know it as a common practice in clinical science that authors give an estimate based on similar studies, so we also would like to act with caution in this point.

Comment 3.8: *As said in lines 169-70 [For SOCRATES and the URICA Precontemplation Scale, we modified the original Likert answer scales into a visual analogue scales to prevent memory biases] can you give more explanation on ‘how modification of the Likert answer scales into a visual analogue scales’ prevent memory biases? (and what these memory biases are)?*

Response: Thank you for pointing out the lack of clarity in this part. Now we use “response biases” instead of “memory biases”. By changing the Likert answer scales into visual analogue scales, we aimed to overcome some disadvantages, such as response biases in repeated measurement studies, which are usually inherent in Likert answer scales, i.e. a respondent remembers that she/he has given a certain answer (i.e. “strongly agree”). Kuhlmann et al. (2016) describe the advantages of visual analogue scales over Likert answer scales in internet-based assessments that are comparable with our online experiment management system, for example that answers are not restricted to a certain number of discrete response options but instead are given in a continuous graphical format which avoids biases in repeated measurement designs.

We added “to prevent response biases due to repeated assessments (i.e. respondents remember their previous answers)” (lines 174 - 175).

Reference:

Kuhlmann T, Reips UD, Wienert J, Lippke S. Using Visual Analogue Scales in eHealth: Non-Response Effects in a Lifestyle Intervention. J Med Internet Res. 2016;18(6): e126. doi: [10.2196/jmir.5271](https://doi.org/10.2196/jmir.5271)

Comment 3.9: *Note that non-parametric parallel of one-way ANOVA is ‘Kruskal-Wallis’ test and not Mann-Whitney test [as said in lines 224-5: If Levene tests revealed heterogeneity of variances, Mann-Whitney tests were used instead.]. Mann-Whitney test is for comparing two groups and parallel to ‘t’ test for two independent groups. In this trial there are two independent groups and application of Mann-Whitney test [when Levene test revealed heterogeneity of variances] is correct. But when there are two independent groups to be compared ‘why apply ANOVA’ which is for comparison of more than two groups? {it is true that ‘if there are only two independent groups ‘ANOVA’s “F” is square of ‘t’ but logic and so algorithm are different}. Whenever there are two independent groups we never apply ANOVA, though ‘F’ and ‘t’ are mathematically similar. Please explain if the reason is what said in lines 231-2 [“We chose ANOVAs as many findings speak for the robustness of the analysis of variance concerning violated assumptions”], {what is that?}.*

Response: You are completely right, and we apologize for the lack of clarity. Regarding violated assumptions, we prefer the ANOVA, because there is strong support for the robustness of the ANOVA under application of non-normally distributed data. To make this clearer, we added this to the sentence in lines 235-237: “We chose ANOVAs as many findings speak for the robustness of the analysis of variance concerning violated assumptions, such as non-normally distributed data.”

We added a reference:

Schmider E, Ziegler M, Danay E, Beyer L, Bühner M. Is It Really Robust? Reinvestigating the Robustness of ANOVA Against Violations of the Normal Distribution Assumption. Methodology. 2010;6(4):147-51.

Thank you for all your constructive comments and suggestions.
